# Circularly Polarized Polariton Lasing from Spin‐Momentum Locking in Deformed Plasmonic Kagome Cavities

**DOI:** 10.1002/adma.202514310

**Published:** 2025-12-09

**Authors:** Zhaoyun Zheng, Chuchuan Hong, Siamak Khorasani, Shreya K. Patel, Marc R. Bourgeois, David J. Masiello, Teri W. Odom

**Affiliations:** ^1^ Department of Chemistry Northwestern University Evanston IL 60208 USA; ^2^ Department of Materials Science and Engineering Northwestern University Evanston IL 60208 USA; ^3^ Department of Materials Science and Engineering University of Washington Seattle WA 98195 USA; ^4^ Department of Chemistry University of Washington Seattle WA 98195 USA

**Keywords:** chiral lasing, exciton‐polariton, plasmonic nanoparticle lattices, surface lattice resonance, symmetry

## Abstract

This paper describes room‐temperature polariton lasing with high circular polarization from plasmonic Kagome lattice cavities strongly coupled to colloidal semiconducting quantum wells. By shrinking and expanding the trimer unit cells in Al nanoparticle lattices, the inversion symmetry is broken, resulting in the spin‐momentum locking of cavity photons. Circularly polarized lattice resonances emerged from crossings of different diffraction orders, denoted as T points, along the Γ‐K direction in momentum space and whose degree of circular polarization is higher than those of high‐symmetry K‐points. Deformed Kagome lattice cavities combined with CdSe nanoplatelets enabled the formation of exciton‐polaritons. Spin‐selectivity from cavity modes resulted in control over the handedness of circular polarization as well as direction of photoluminescence from lower polaritons. Six lasing beams from T points has alternating handedness, low thresholds (8 µJ cm^−^^2^), and high degrees of circular polarization (≈0.7). The anticipation that spin‐momentum locking via plasmonic Kagome lattices can be extended to other non‐Bravais metasurfaces with hexagonal symmetries and opens prospects for exploring optical analogues of spin and valley physics.

## Graphical abstract

This paper describes room‐temperature polariton lasing with high circular polarization from deformed plasmonic Kagome lattice cavities strongly coupled to colloidal CdSe nanoplatelets. Spin‐selectivity from cavity modes resulted in control over the handedness of circular polarization as well as the direction of polariton lasing, opening prospects for exploring optical analogues of spin and valley physics.

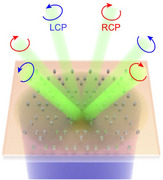

## 1 Introduction

Exciton‐polaritons are quasi‐particles formed through strong coupling between cavity photons and excitons, exhibiting properties from both constituents. In particular, the spin degree of freedom has generated emerging applications in quantum information and optoelectronic technologies.^[^ ^1^, ^2^, ^3^ ^]^ In polariton lasing or condensation, coherent states can enable fast information read‐out and long‐range energy transport advantageous for spin transport over macroscopic distances.^[^ ^4^, ^5^, ^6^ ^]^ The most common approach to manipulate polariton spin is through the excitonic component using intrinsic spin‐momentum locking in electronic materials having non‐centrosymmetric crystal structures. Although exciton‐polaritons are responsive to circularly polarized light with opposite handedness, external magnetic fields and cryogenic temperatures are required to create large nonequilibrium exciton spin populations and to suppress spin flipping.^[^ ^7^, ^8^, ^9^, ^10^ ^]^ Alternatively, spin angular momentum can be engineered in optical nanostructures, represented as either left or right‐circular polarization (LCP or RCP). In the optical Rashba‐Dresselhaus (ORD) effect, which describes spin‐dependent photon scattering, LCP and RCP light are deflected to wavevectors of equal magnitude but opposite directions in momentum space to maintain spin‐momentum conservation.^[^ ^11^, ^12^, ^13^ ^]^ Because photon spin is minimally affected by thermal fluctuations,^[^ ^3^, ^14^, ^15^ ^]^ unlike electrons, manipulation is possible at room temperature.

In Fabry‐Pérot cavities conventionally used to form exciton‐polaritons, ORD effects have been induced by birefringent liquid crystals.^[^ ^2^, ^3^, ^16^ ^]^ However, since an applied external voltage is needed to reorient the anisotropic molecules, the quality of the cavity resonance is compromised. Optical metasurfaces have also exhibited ORD effects by breaking inversion symmetry of the cavities via tailoring the size, shape, and/or arrangement of unit cells.^[^ ^17^, ^18^, ^19^, ^20^, ^21^, ^22^ ^]^ For example, 2D square lattices of triangular nanostructures or nanorod pairs with different orientations can support circularly polarized modes that are typically located near the Γ‐point of the first Brillouin zone.^[^ ^18^, ^23^, ^24^, ^25^ ^]^ Generating chiral modes away from the Γ point is important for expanding the angular range as well as directional control over circularly polarized emission. Optical metasurfaces with hexagonal symmetries, like honeycomb or Kagome lattices, exhibit high‐symmetry K and K′ points related by inversion and time‐reversal symmetry.^[^ ^26^, ^27^ ^]^ Breaking such symmetry lifts the equivalence and results in spin‐selective modes^[^ ^11^, ^28^ ^]^ that are analogous to spin‐valley selectivity in transition metal dichalcogenides.

Plasmonic nanoparticle lattices are a promising platform to study photonic non‐Bravais lattices with hexagonal symmetries for strong light‐matter coupling. The hybridization of localized surface plasmons of individual nanoparticles with diffractive orders of the lattice can form surface lattice resonances (SLRs).^[^ ^29^, ^30^ ^]^ In contrast to their dielectric counterparts, SLRs provide ultra‐small mode volumes because of the plasmonic nature, and comparably high quality factors from the collective resonance.^[^ ^31^, ^32^ ^]^ Integration of lattice cavities with high oscillator‐strength semiconducting emitters, such as CdSe nanoplatelets, has enabled room‐temperature polariton lasing with properties tunable by unit‐cell engineering.^[^ ^33^, ^34^, ^35^, ^36^, ^37^ ^]^ Compared with square lattices,^[^ ^33^, ^38^ ^]^ hexagonal lattices have six‐fold rotational symmetry that can enable non‐orthogonal coupling between unit cells and effectively modulate the spin states of the scattered photons. For example, plasmonic nanoparticle Kagome lattices with deformed trimer unit cells can support SLR modes selective for circular polarization at high‐symmetry K points.^[^ ^28^ ^]^ Since non‐Bravais plasmonic lattices contain multiple scattering elements per unit cell, they offer design flexibility to modify the shape and polarization of photonic band structures by near‐, intermediate, and far‐field coupling among nanoparticles.^[^ ^35^, ^36^, ^37^, ^39^, ^40^, ^41^ ^]^ Understanding how lattice geometry and unit cell modifications affect polariton lasing emission will provide important insight for controlling the spin degree of freedom in polaritonic systems.

Here we demonstrate spin‐selective polariton lasing from deformed plasmonic Kagome lattices strongly coupled to CdSe nanoplatelets. By breaking the Kagome lattice symmetry in real space, we lifted the degeneracy of SLR modes at high‐symmetry K points as well as at T points along the Γ‐K direction in momentum space. We observed ORD effects in SLR modes located at opposite in‐plane momentum, each having circular polarization with opposite handedness. Coupled dipole calculations revealed that the origin of spin‐momentum locking of cavity photons was from near‐field electric dipole rotations protected by time‐reversal symmetry. We demonstrated that the spin‐dependent characteristics of the lattice cavities could be transferred to exciton‐polaritons by strong coupling with 4‐monolyaer CdSe nanoplatelet films. Under optical pumping, the system exhibited lasing from the lower polariton band with a characteristic six‐fold pattern in momentum space corresponding to the hexagonal lattice symmetry. The polariton lasing beams showed alternating circular polarization handedness with high LCP and RCP contrast (degree of circular polarization ≈0.7). Spin‐selective separation of polaritons enabled by deformed Kagome plasmonic lattices offers prospects for spin‐optoelectronic devices at room temperature and without magnetic fields.

## 2 Results and Discussion

**Figure 1:**
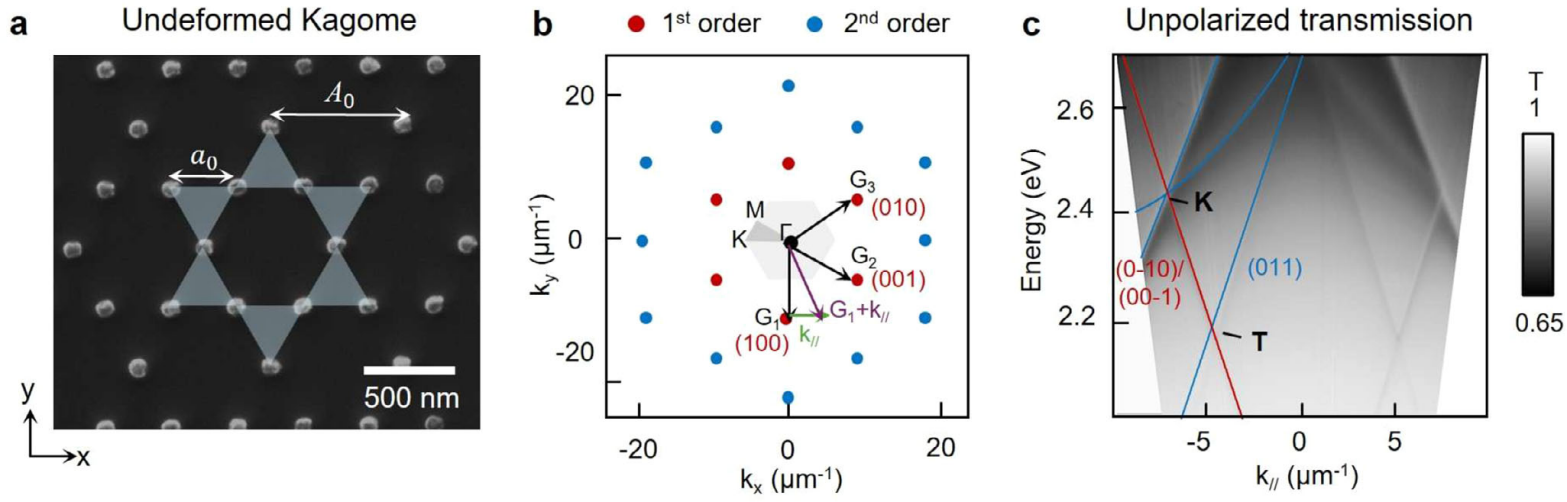
K and T points in undeformed plasmonic Kagome lattices. a) SEM image of the fabricated Al NP lattice. The blue triangles indicate trimer unit cells; white arrows denote periodicity (*A*_0_) and interparticle spacing (*a*_0_). b) Reciprocal space of the undeformed lattice. The grey hexagon denotes the first Brillouin zone with high‐symmetry points Γ, M, and K labeled. Black arrows indicate reciprocal lattice vectors G_1_, G_2_, and G_3_; the in‐plane wavevector *k_∥_* is highlighted in green, and purple vector shows the addition of in‐plane *k_∥_* to the diffractive orders G_1_G₁. The 0th, 1st and 2nd diffractive orders are shown in black, red, and blue dots, respectively. c) Angle‐resolved transmission along Γ–K under unpolarized illumination for *A*_0_ = 580 nm lattice, with K and T points labeled. The T point is from crossing of (011) and (0–10)/(00–1) modes. The empty lattice approximation dispersion is overlaid on the measurement.

A prerequisite to induce spin‐momentum locking in non‐Bravais lattices is breaking inversion symmetry. **Figure** **1**a first establishes properties of undeformed plasmonic Kagome nanoparticle lattices at different symmetry points and directions in reciprocal space. We fabricated Al Kagome lattices with trimer unit cells (interparticle spacing *a*_0_) in a hexagonal pattern (lattice constant *A*_0_). *A*_0_ was set to 580 nm so that SLR modes were at visible wavelengths in a uniform refractive index environment *n* = 1.52. Figure 1b depicts the reciprocal space representation of the Kagome lattice, where the diffractive orders are represented by black, blue and red dots. The three in‐plane basis vectors (G_1_, G_2_, G_3_) are represented by arrows, separated by 60°, and labeled as (100), (010), and (001), respectively. The photonic in‐plane dispersion is | *k_//_* + *G_//_* | = |*k*_inc_|, where *k*_//_ is the in‐plane incident wavevector, *G*_//_ is a reciprocal lattice vector defined by *A*_0_, and *k*_inc_ = *n*_eff_ *E* / ℏ*c* is the incident photon wavevector with energy *E* in an effective refractive index *n*_eff_. Figure 1c displays the measured angle‐resolved transmission under unpolarized light overlaid with calculated empty lattice dispersion bands along the Γ‐K direction. Both the measurement and calculation exhibited a crossing of three sets of diffractive modes at *k*_//_ ≈ ±7.0 µm^−1^ and *E* ≈ 2.43 eV, identified as the K point, as well as a crossing at *k*_//_ ≈ ±4.7 µm^−1^ and *E* ≈ 2.17 eV, referred to as the T point.^[^ ^42^ ^]^ For our system, the T point is defined by the intersection of (011) and (0‐10)/(00‐1) diffraction orders.

**Figure 2:**
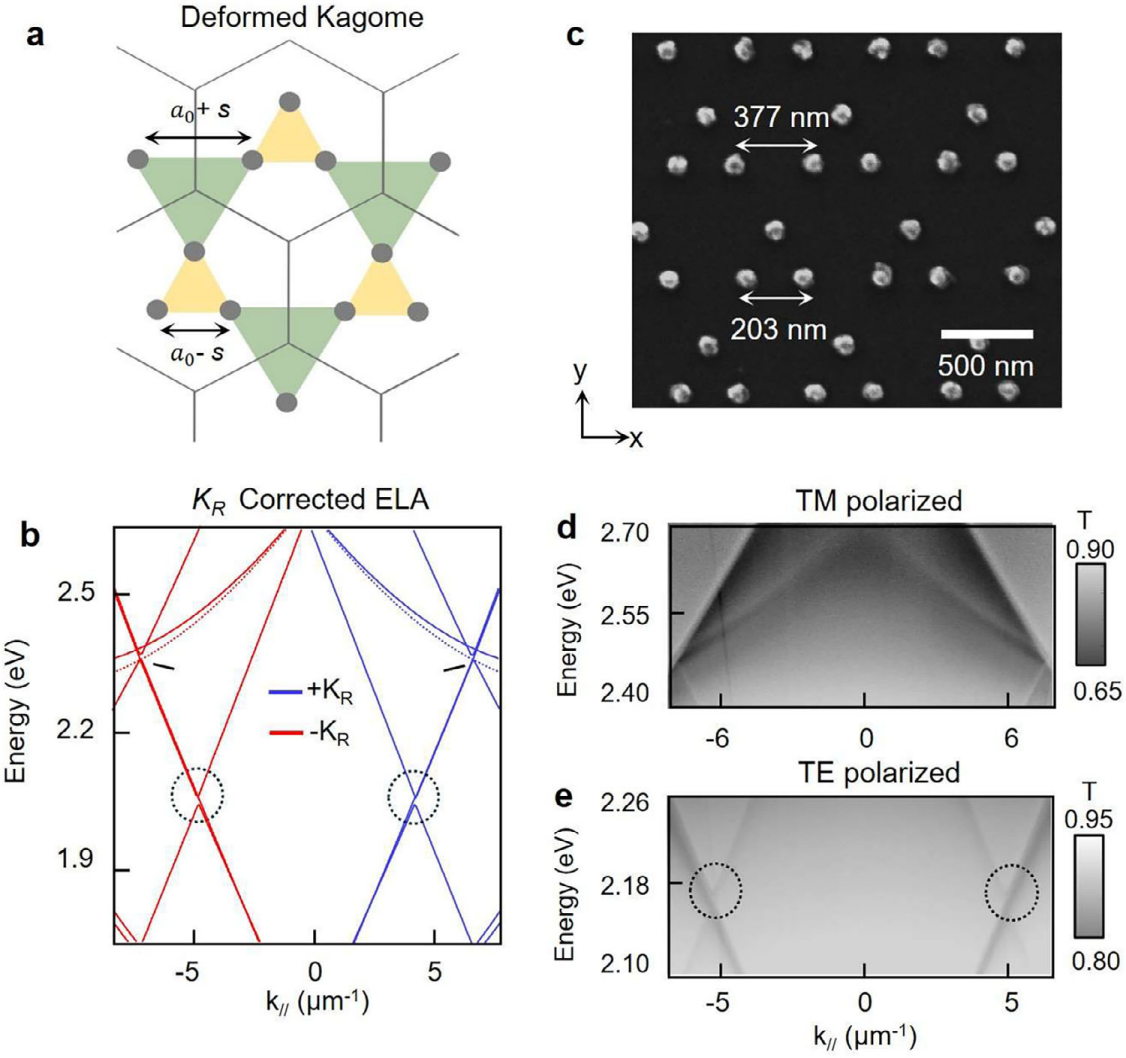
Deformed Kagome lattices exhibit mode splitting at K and T points. a) Scheme of the deformed lattice. Yellow and green triangles indicate shrunken (*a*_0_ ‐ *s*) and expanded (*a*_0_ + *s*) trimer unit cells, respectively. b) *K_R_* ‐corrected empty lattice dispersion for an *A*_0_ = 580 nm lattice. Mode splitting was observed at K and T points (with ‐ *K_R_*) and K′ and T′ points (with + *K_R_*). (c) SEM image of a fabricated Al nanoparticle lattice with *a*_0_ ‐ *s* = 203 nm and *a*_0_ + *s* = 377 nm. (d) TM‐polarized angle‐resolved transmission shows a pair of parabolic bands along the along Γ–K direction. (e) TE‐polarized transmission shows band gaps opening at the T points (dotted circles).

Although inversion symmetry can be broken by incorporating nanoparticles having different diameters or by varying the orientation of nanorods within the unit cell,^[^ ^12^, ^43^ ^]^ strict fabrication constraints are required. In contrast, deformations based on shrinking and expanding trimer nanoparticle unit cells are intuitive and rely only on the parameter *s*, the degree of length change. Keeping the hexagonal lattice spacing *A*_0_ constant, the shrunken unit cell is defined as *a*_0_ – *s* (**Figure** **2**a, yellow triangles) and the expanded unit cell as *a*_0_ + *s* (Figure 2a, green triangles). To determine how symmetry breaking in real space affects the band structure in momentum space, we incorporated *K_R_*, a spin‐dependent geometric correction^[^ ^44^ ^]^ in the empty lattice approximation, represented by the equation | *k_//_* + *G_//_* ± *α_R_K_R_* | = |*k*_inc_|. This modified *G*′ contains a distortion resulting in a global rotation of the reciprocal lattice, either clockwise or counterclockwise, depending on the sign of *K_R_*. The degree to which *K_R_* influences the reciprocal lattice vectors G_i_ is directly correlated with the extent of deformation (i.e., magnitude of *s*) and weighting parameter *α_R_* (Figure S1, Supporting Information).

Figure 2b depicts the calculated dispersion, where a pair of parabolic bands intersect two linear bands at the K points. The solid parabolic band follows the dispersion of the undeformed Kagome lattice, while the lower, dashed band emerges from unit‐cell deformation; the linear bands crossing at the K point open up a bandgap.^[^ ^28^ ^]^ Notably, a discontinuity in the SLR dispersion also occurs at the T points (dotted circles), which provides a more straightforward route to control interactions between two SLR modes for spin‐momentum locking compared with the three sets of diffraction orders at the K‐points. Similar modifications in dispersion are also observed at the K′ and T′ points, which are degenerate in energy with the K and T points, respectively, but are at opposite wavevectors. Finite‐difference time‐domain (FDTD) simulations were performed to confirm mode splitting at the K and T points (Figure S2, Supporting Information). Sweeping *s* from 0 to 100 nm revealed that the intensities of the split modes were most clearly defined when *a*_0_ – *s* = 203 nm and *a*_0_ + *s* = 377 nm; hence, we used these parameters when fabricating the deformed Kagome lattices (Figure 2c).

**Figure 3:**
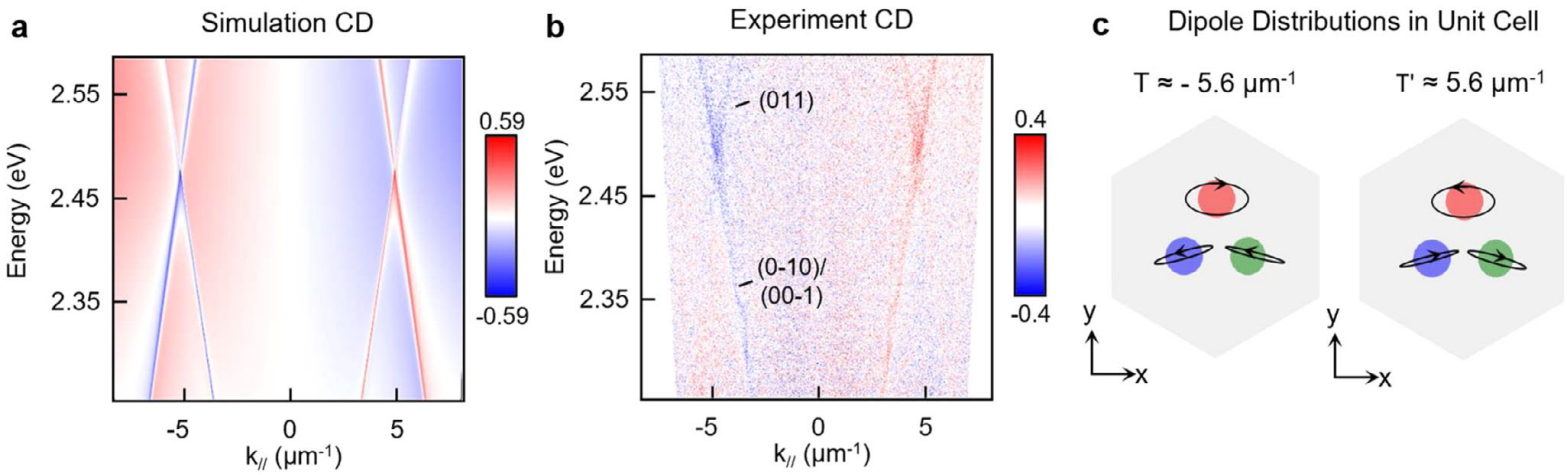
Spin‐momentum locking of deformed plasmonic Kagome lattice at T points. Circular dichroism (CD) transmission of a) FDTD simulations and b) measured transmission shows spin‐split dispersion near the T points of Al nanoparticle lattice (*A*_0_ = 500 nm, *a*_0_ ‐ *s* = 175 nm, *a*_0_ + *s* = 325 nm, *d* = 70 nm and *h* = 55 nm) in an index‐matched environment (*n* = 1.525). c) SLR eigenmodes displayed within a single trimer unit cell show opposite rotation directions at the T and T′ points, as demonstrated by the black orbitals and arrows on each sublattice. The elliptical dipole distribution and the opposite dipole rotation at each nanoparticle site between the T and T′ points indicate the presence of spin‐momentum locking.

To resolve this splitting experimentally, we measured the optical transmission of deformed Al Kagome lattices in a uniform refractive index environment (*n* = 1.52). Under transverse magnetic (TM)‐polarized illumination, two sets of parabolic bands were present along the Γ–K direction, in agreement with the *K_R_*‐corrected empty lattice approximation (Figure 2d). Under transverse electric (TE)‐polarized illumination, an avoided crossing appeared at the T points with an energy splitting of ≈30 meV (Figure 2e, dotted circles). Reducing the rotational symmetry of the lattice from 6‐fold (Figure 1a) to 3‐fold (Figure 2c) lifted mode degeneracy at the K points and introduced mode splitting at the T points.

By adjusting *A*_0_, we selectively coupled different lattice cavity modes to heavy‐hole excitons of CdSe nanoplatelets. To optimize interactions between SLR modes at the T point and Wannier‐Mott excitons with absorption at 2.42 eV, we designed a Kagome lattice with a smaller periodicity (*A*_0_ = 500 nm, *a*_0_ = 250 nm) and scaled deformation (*s* = 75 nm) (Figure S3, Supporting Information). FDTD calculations based on experimental parameters confirmed that: 1) the band‐edges of T‐points were shifted to *k_//_* = ±5.6 µm^−1^ and *E* = 2.51 eV, close to the exciton resonance; 2) the circular dichroism (CD) of the transmission band structure showed a spin‐selective response the T points (**Figure** **3**a). CD is defined as the difference in transmission intensities under LCP and RCP plane‐wave excitations normalized by their sum. The simulation revealed that bands at the T (*k*_//_ = ‐5.6 µm^−1^) and the T′ point (*k*_//_ = +5.6 µm^−1^) have CD values of ±0.59. Compared with modes near the K and K’ points (Figure S4, Supporting Information), the T points exhibited ≈2x increase in the overall CD magnitude.

Figure 3b indicates that the measured CD of a deformed Kagome lattice under index matched conditions agrees well with numerical simulations. We note that the first‐order (0–10) and (00–1) modes have higher transmission intensity compared to the second‐order (011) mode since higher‐order diffraction modes have both reduced scattering and lower absorption cross‐sections. This difference in transmission results in elliptically polarized far‐field profiles since, for pure circularly polarized modes, intersecting bands need to be of equal intensity and exhibit a phase difference of π/2.^[^ ^45^, ^46^ ^]^

To understand the origins of opposite circular polarization at the T and T′ points, we examined the SLR eigenmodes of the deformed Kagome lattice based upon a coupled dipole approximation model^[^ ^47^ ^]^ (Figure 3c). The black orbital traces show the precession of electric dipoles on each nanoparticle within the trimer unit cell, with dipole rotations at T points opposite to those at T′ points. This reversal in handedness in the local dipole rotation indicates that spin‐momentum locking is governed by time‐reversal symmetry, which here is only partially preserved due to intrinsic losses in plasmonic systems.The orbital traces at each nanoparticle have different magnitudes because the T points lack the three‐fold rotational symmetry necessary for valley pseudo‐angular momentum to be well‐defined.^[^ ^48^, ^49^ ^]^ Near‐field calculations reveal strong field confinement at the nanoparticle surfaces; the elliptical dipole polarization on each nanoparticle site occurs only in the deformed lattice because of broken‐inversion symmetry, in agreement with group‐theory predictions (Figure S5, Supporting Information). In contrast, undeformed Kagome lattices exhibit only linear dipole distributions at the T points, which indicates that the SLR mode carries no spin polarization (Figure S6, Supporting Information).

**Figure 4:**
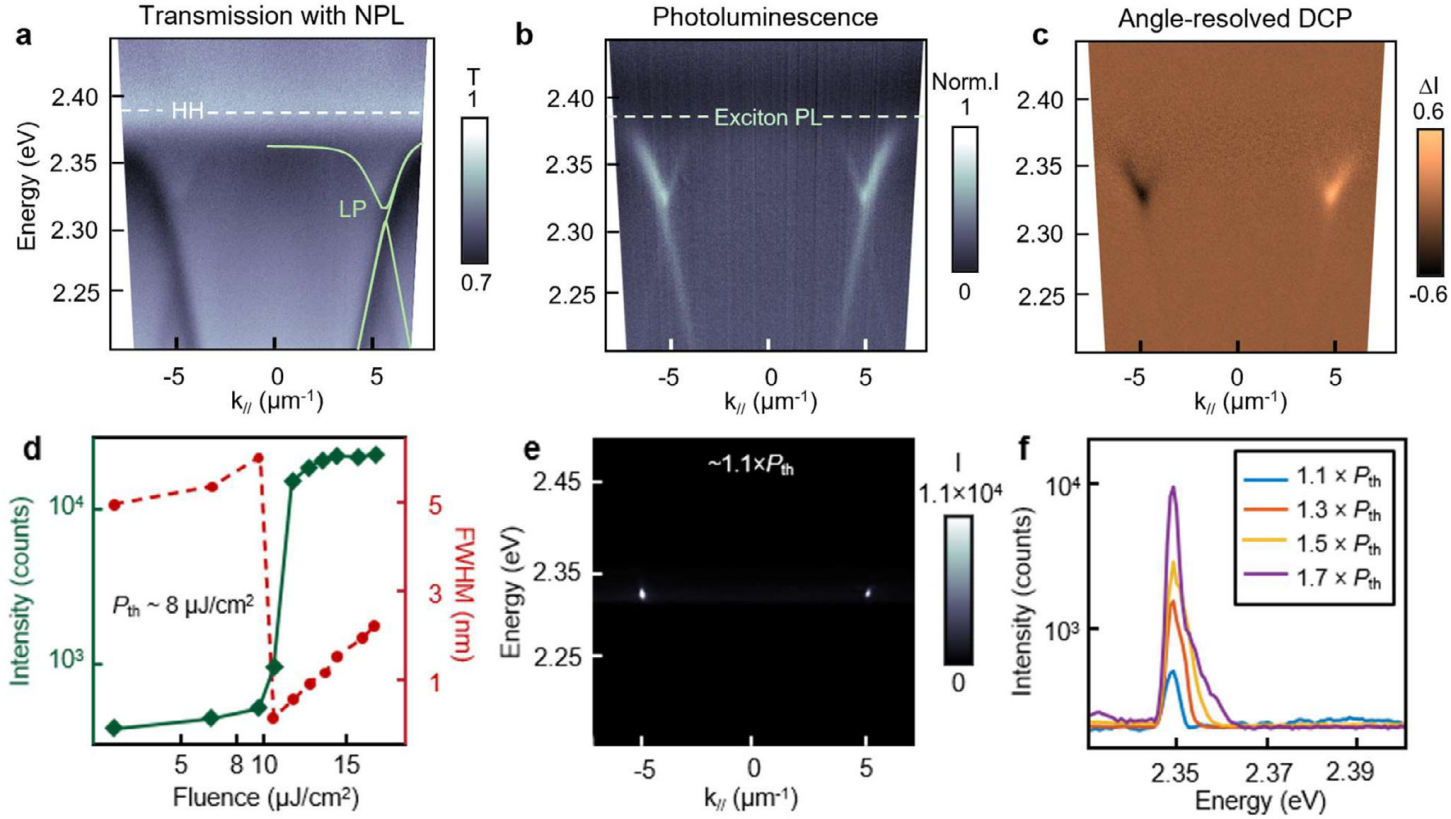
Exciton‐polaritons from T points strongly coupled to CdSe nanoplatelets. a) The optical transmission spectrum shows band bending near the T points. Green lines illustrate lower polariton bands from the coupled‐oscillator model. b) Bright lower polariton (LP) photoluminescence emerges at the T and T′ points. c) DCP in the angle‐resolved photoluminescence confirms circularly polarized emission from the LPs. d) The light‐light curve exhibits a superlinear increase in intensity (green curve) and a sudden linewidth narrowing (red dashed line) at the lasing threshold of 8 µJ cm^−^^2^. Beyond the lasing threshold, the linewidth starts to broaden. e) Angle‐resolved lasing spectra shows emission at the T and T′ points. f) Spectra above threshold shows blue‐shifting and broadening of the polariton lasing wavelength as the pump fluence increases.

To investigate how non‐centrosymmetric cavities influence polarization of photoluminescence, we combined deformed Kagome Al plasmonic lattices with high‐oscillator strength 4‐monolayer CdSe nanoplatelets (Experimental Section). The absorption spectrum of the CdSe nanoplatelet film showed that the heavy‐hole exciton had a sharp transition at 2.42 eV with a linewidth ≈50 meV (Figure S7, Supporting Information). **Figure** **4**a indicates that the system is strongly coupled based on band bending below the heavy‐hole energy. Green curves overlaid on the dispersion diagram are eigenmode calculations for the lower polariton branch based on the coupled‐oscillator model with a Rabi splitting of 75 meV, which is larger than the average linewidths of the cavity (18 meV) and exciton resonance (Figure S8, Supporting Information). Due to the refractive index mismatch between the 110‐nm nanoplatelet film (*n* = 1.83) and glass substrate (*n* = 1.52), waveguiding effects, which were also confirmed by the phase flip at the film‐air interface in FDTD calculations (Figure S9, Supporting Information), resulted in a red‐shifting of T point band edges by 0.19 eV. Under fs‐laser excitation at low fluences (5 µJ cm^−^^2^), energy was transferred from the flat exciton bands (at 2.42 eV) to the dispersive lower polariton states.

Angled‐resolved photoluminescence shows clear lower polariton emission after normalizing and subtracting the pristine film signal; the intensity is highest at the T‐point band edges (*k*_//_ = ±5.6 µm^−1^, *E* = 2.33 eV; white arrows) due to the high photon density of states (Figure 4b). Additionally, emission was brighter at in‐plane momenta beyond the T and T′ points since first‐order diffraction modes have higher transmission than second‐order modes. Figure 4c shows the degree of circular polarization (DCP), defined as (LCP − RCP) / (LCP + RCP), reached ±0.6, with LCP (RCP)‐dominated emission at the T (T′) point. Since CdSe nanoplatelets are randomly oriented within the spin‐cast film and lack a preferred emission polarization, the circular polarization of the polariton emission is dictated by the cavity modes; the DCP maps in Fourier space exhibit the same values as in angle resolved PL measurements (≈±0.6) (Figure S10, Supporting Information).

To achieve polariton lasing emission, we increased the pump power and measured a nonlinear rise in photoluminescence intensity that was also accompanied by abrupt linewidth narrowing at a threshold (*P*_th_) of 8 µJ cm^−^^2^ (Figure 4d). This low threshold is comparable to polariton lasing from plasmonic lattices and CdSe nanoplatelet films.^[^ ^33^, ^34^, ^50^, ^51^ ^]^ Below *P*_th_, the emission linewidth was broad (>5 nm); at ≈*P*_th_, the reduction in spectral linewidth from T‐point emission can be associated with the buildup of coherence in lower polariton emission.^[^ ^52^, ^53^ ^]^ Figure 4e demonstrates that just above the lasing threshold (1.1 × *P*_th_), the lower polariton emission condenses into their respective band minima (*k*_//_ = ±5.6 µm^−1^ and *E* = 2.345 eV) due to polariton‐stimulated scattering. At higher pump fluences, the central wavelength of the lasing emission blue‐shifted and broadened from 2.34 to 2.35 eV (Figure 4f) from repulsive polariton‐polariton and polariton‐exciton interactions.^[^ ^38^, ^54^, ^55^, ^56^ ^]^ The angle‐resolved lasing beams confirm that the blue‐shifted, bright emission spots are exclusively from the T points because of the high quality factor at the band edges (Figure S11, Supporting Information).

**Figure 5:**
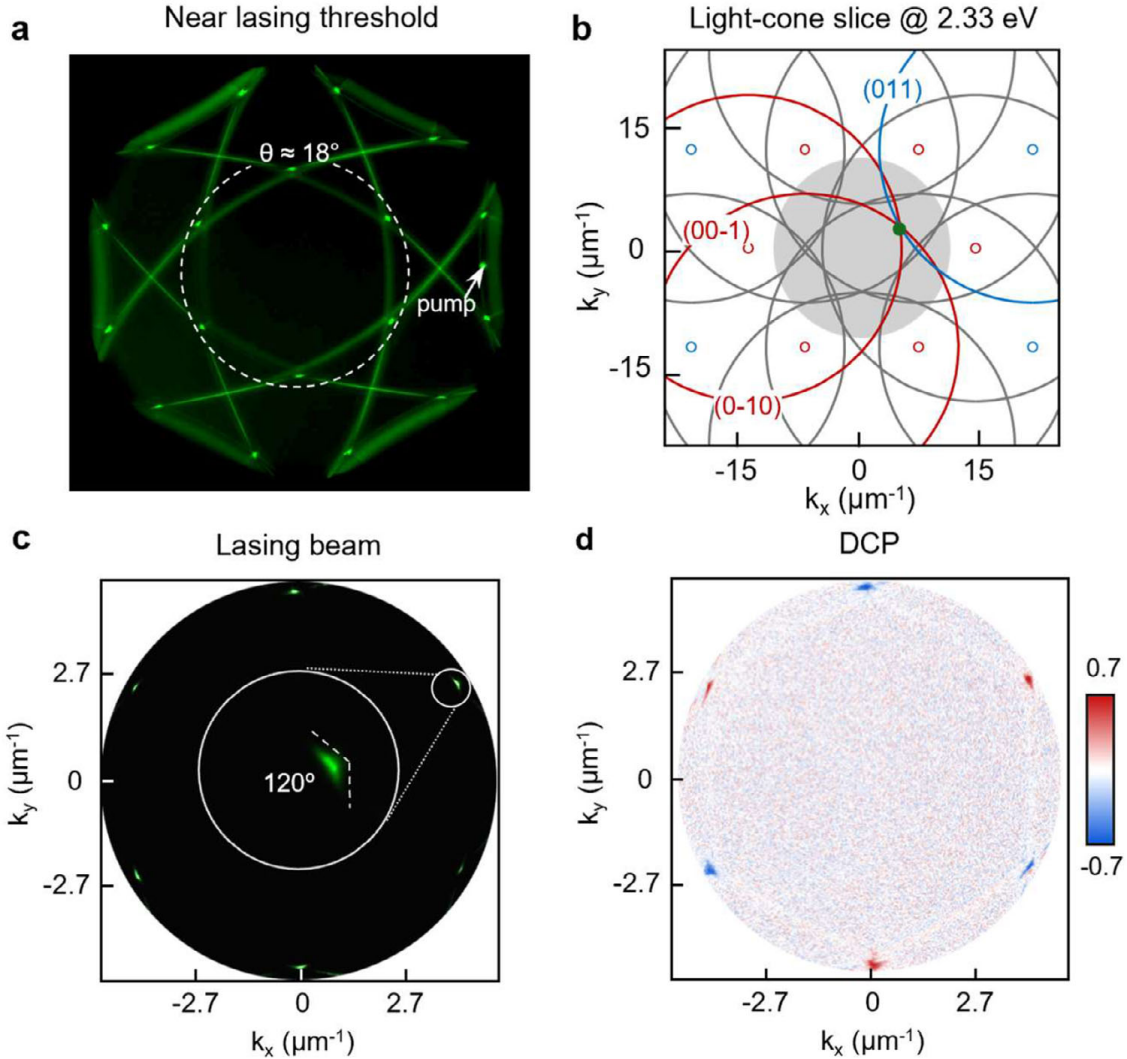
T‐point polariton lasing from deformed plasmonic Kagome lattices. a) Measured back focal plane image near the lasing threshold (*P*_th_) shows an inner hexagonal emission pattern at the polar angle of θ ≈ 18°(dashed circle). b) 2D slice of a light cone model at 2.33 eV, the lasing energy from T‐point lasing band edge. T points are formed at the intersections of two red and one blue isoenergetic contours. The gray circle indicates the numerical aperture of the objective lens used for measurements. c) Clear lasing emission from all six T points are observed at 1.5 × *P*_th_. The angular range is cropped to highlight only the T‐point lasing pattern. Each lasing beam is a boomerang shape with internal angle of 120°. d) High DCP of 0.7 confirms that the spin‐selectivity from six T points can efficiently control the circular polarization of output lasing emission.

Near polariton lasing threshold (≈*P*_th_), the back focal plane image showed a hexagonal pattern with vertices around a polar angle (θ) of 18° from the surface normal (**Figure** **5**a). At higher θ, six cross‐shaped patterns and twelve bright spots were present. Because lasing emission from the T points was ≈±18° (Figure 4e), we analyzed the inner hexagonal pattern. To understand intuitively the momentum‐space distribution, we used a model based on light cones centered about diffraction orders and evaluated the in‐plane scattered light at the energy associated with lasing (Figure 5b). With circular cross sections having radii given by the T‐point band edges (2.33 eV), we confirmed that two first‐order modes crossing one second‐order diffractive mode contributed to the lasing emission. For example, (0–10) and (00–1) (red circles) intersected with (011) (blue circle), with the crossing point defining a T point (green dot). The additional six cross‐shaped patterns and twelve emission spots at larger polar angles in Figure 5a can also be explained by the intersections of distinct first‐ and second‐order diffraction modes (Figure S12, Supporting Information).

**Figure** **5**c presents a zoomed‐in back Fourier plane image of lasing emission above threshold (1.1 × *P*_th_) near the T points (θ ≈ 18°). The momentum‐space positions of these beams can be associated with the reciprocal hexagonal structure defined by the T and T′ points, with beam shapes slightly elongated about the vertices with an internal angle ≈120°. Since T points are not high‐symmetry points, optical feedback is from interference between intersecting in‐plane propagating modes, which broadens the spatial profile of the emission beam,^[^ ^35^, ^57^ ^]^ in contrast to the biaxially confined lasing from K points (Figure S13, Supporting Information).

Figure 5d shows the DCP map of the six lasing beams with similar amplitudes and alternating handedness. Red spots represent the helicity of the T points (LCP), while blue spots represent that of the T′ points (RCP); the measured DCP values were ± 0.7. The flip in handedness of polariton lasing emission every 60° is directly associated with three‐fold rotational symmetry of the cavity. We also measured the DCP of T‐point lasing from undeformed lattices that was low (<0.1) and did not exhibit circularity in the lasing emission (Figure S14, Supporting Information). Hence, as expected, the SLRs are predominately linearly polarized when inversion symmetry of the Kagome lattice is preserved, but breaking this symmetry results in spin‐dependent lattice plasmon modes that enable efficient generation of LCP and RCP beams with highly directional, off‐normal emission.

## 3 Conclusion

In summary, we realized room‐temperature polariton lasing with high circular polarization resulting from spin‐momentum locking of deformed Kagome lattices. The measured DCP (0.7) was relatively high compared to other chiral polariton systems.^[^ ^33^, ^58^, ^59^, ^60^ ^]^ Our results highlight that by breaking inversion symmetry via real‐space lattice deformation, circularly polarized modes can emerge not only at high‐symmetry K points but also along Γ– K directions (T points), where even stronger chiroptical responses were possible. Under optical pumping, high‐gain semiconducting nanomaterials combined with symmetry‐broken plasmonic lattice cavities resulted in circularly polarized polariton lasing; the six‐fold emission beam profiles had alternating handedness, with three LCP beams associated with T points and three RCP beams corresponding to T′ points. This spin angular momentum‐dependent selectivity is analogous to spin‐valley physics in electronic materials. We anticipate deformed non‐Bravais hexagonal lattices will allow effective control over gain and loss and result in a versatile nanophotonic platform to explore parity‐time symmetry and topological effects.

## 4 Experimental Section

### Synthesis of 4‐Monolayer CdSe Nanoplatelets (NPLs)

CdSe NPLs were synthesized based on a previously reported method.^[^ ^61^ ^]^ The Cd(Myristate)_2_ precursor was prepared by adding 3 g of Cd(NO_3_)_2_·4H_2_O dissolved in 200 mL of methanol to a solution of 5 g of Na_2_(Myristate) in 500 mL of methanol. The mixture was stirred at room temperature overnight. The resulting precipitate was collected by vacuum filtration, washed three times with methanol, and then dried under vacuum overnight.

Then, in a three‐neck round‐bottom flask, 850 mg of Cd(Myristate)_2_, 130 mg of Se powder, and 75 mL of octadecene were degassed and reacted under vacuum at 95 °C for 30 min. The temperature was then raised to 240 °C under a steady nitrogen flow. When the mixture reached 205 °C, 200 mg of Cd(Ac)_2_·2H_2_O was added. The reaction continued at 240 °C for 8 min before rapid cooling under nitrogen. Once the temperature dropped to 180 °C, the flask was placed in a water bath. At 160 °C, 2.5 mL of oleic acid was added to stabilize the colloidal CdSe NPLs, and the solution was cooled to room temperature.

To purify the NPL solution, the product was first divided into four centrifuge tubes, each with 20 mL of n‐hexanes each. After centrifugation of the tubes at 3000 rpm for 5 min, the supernatant was discarded. The resulting pellets were redispersed in hexanes and centrifuged again at 10 000 rpm for 5 min, and the supernatant was collected and concentrated to ≈12 mL. The concentrated solution was then washed with methyl acetate and centrifuged at 10 000 rpm for another 5 min. The final precipitate was redispersed in cyclohexanes to a concentration of 60 mg mL^−1^ for subsequent experiments.

### Sample preparation

We fabricated aluminum nanoparticle (Al NP) Kagome lattices on glass substrates using electron‐beam lithography (EBL, Raith Voyager 100). To prepare the substrate for patterning, a thin layer of PMMA A3 photoresist was spin‐coated onto the glass surface. 7‐nm of gold was thermally evaporated onto the glass prior to patterning to reduce the charging effects from the insulating substrate. The desired lattice structures were written into PMMA by EBL, followed by development in a 1:3 mixture of MIBK (methyl isobutyl ketone) and IPA (isopropanol) to form a nanohole mask. Aluminum was then deposited through the mask via electron‐beam evaporation, and lift‐off was carried out acetone to remove the remaining photoresist.

### Angle Resolved Transmission Measurements

The photonic band structures of the plasmonic lattices were characterized using a Fourier microscopy setup. The back focal plane of a 20× Plan Apo objective lens (Nikon Japan, NA = 0.75) was projected onto the entrance slit of a SP2500 spectrometer (Teledyne Princeton Instruments). This objective collected scattered light from the sample over an angular range of ≈ ±48°. Angle‐ and wavelength‐resolved images were recorded using a CCD camera (PIXIS 400, Teledyne Princeton Instruments). To convert the measured data from wavelength (λ) and incident angle (θ) to energy (*E*) and in‐plane momentum (*k_/_*_/_), we applied the following equations: E = hc/λ and k_//_ = (2πn/λ) sin θ. Linearly polarized lattice dispersion measurements were performed by placing a linear polarizer directly after the white light source to control incident polarization. Transverse electric (TE) and transverse magnetic (TM) polarizations correspond to electric and magnetic fields oriented parallel to the 2D lattice plane, respectively. When transmission spectra is collected along the *k_x_* direction, as defined by the orientation of the entrance slit, the electric (magnetic) field of the incident light is aligned along the *k_y_* direction for TE (TM) polarization. Circularly polarized light was produced by combining a linear polarizer with a quarter‐wave plate (QWP). The fast axis of the QWP and the linear polarizer were set to 45° or –45° to selectively generate left‐ and right‐handed circularly polarized illumination, respectively. To maintain a uniform refractive index environment around the nanoparticle lattice, a drop of immersion oil (*n* = 1.52) was applied to the sample and sealed with a glass coverslip.

### Photoluminescence and Lasing Measurements

60 µL of CdSe NPLs (60 mg mL^−1^) were spin‐cast on Al Kagome lattices at 3000 rpm to form a uniform film. Samples were pumped using a pulsed Ti:sapphire laser (100 fs pulse duration, 420 nm wavelength, 1000 Hz repetition rate). Angle‐resolved photoluminescence was collected using the same Fourier microscopy setup as the transmission measurements. A 450 nm long‐pass filter was placed after the sample to block pump signals, and neutral density filters were applied to reduce lasing beam intensity to avoid detector saturation. The circular polarization of the output emission was analyzed by placing an achromatic quarter‐wave plate followed by a linear polarizer in the collection path. For capturing the back focal plane image of the lasing emission, the entrance slit of the spectrometer and center grating were removed so that the collected light can be directly projected onto the CCD camera.

### FDTD Simulations

Finite‐difference time‐domain (FDTD) simulations (Ansys‐Lumerical) were conducted to model the linear optical response of Al NP lattices, with optical constants for Al from the Palik handbook. Optical dispersions under TE and TM polarizations were calculated by simulating transmission at varying incident angles along the Γ–K direction with a BFAST plane wave source. To compute field profiles for waveguide mode simulations, a 110‐nm‐thick dielectric slab with a refractive index *n* = 1.80 was placed over the lattice, with air (*n* = 1) as the surrounding medium above the film.

### Coupled Dipole Approximation

Within the coupled dipole approximation,^[^ ^30^ ^]^ nanoparticles are represented by polarizable electric dipole moments that self‐consistently interact with each other as well as with any external driving fields. For 2D lattices with *N_κ_* sites per unit cell, the adoption of Bloch mode solutions leads to a (*3N_κ_* × 3*N_κ_*) matrix problem ${\bar{\Pi }}_{k_{∥}}^{-1}\left(\omega \right)p_{k_{∥}}=E_{k_{∥}}^{0}$. The vectors $E_{k_{∥}}^{0}$ and $p_{k_{∥}}$ describe the Bloch waveincident field and induced dipole moments, respectively, associated with in‐plane wavevector $k_{∥}$, and the matrix ${\bar{\bar{\Pi }}}_{k_{∥}}^{-1}\left(\omega \right)$ accounts for intra‐ and inter‐sublattice electromagnetic coupling. Dispersive SLR Bloch eigenmodes arise at $[k_{∥},\omega_{\lambda }(k_{∥})]$ points where the coupling matrix becomes non‐invertible. Complex‐valued SLR eigenfrequencies $\omega_{\lambda }(k_{∥})$ and the corresponding SLR eigenmode polarization vectors $\varepsilon_{k_{∥}}^{\lambda }[\omega_{\lambda }(k_{∥})]$ representing non‐trivial solutions to ${\bar{\Pi }}_{k_{∥}}^{-1}\left(\omega \right)\varepsilon_{k_{∥}}^{\lambda }=0$ were identified numerically.^[^ ^47^ ^]^ The far‐field polarization states of light radiated into given directions by SLR eigenmodes $\varepsilon_{k_{∥}}^{\lambda }$ were also computed. A detailed mathematical formulation of the model is provided in the S15 (Supporting Information).

## Conflict of Interest

The authors declare no conflict of interest.

## Supporting information



Supporting Information

## Data Availability

The data that supports the findings of this study are available from the corresponding author upon reasonable request.
