# Supplementary material for: Circularly Polarized Polariton Lasing from Spin‐Momentum Locking in Deformed Plasmonic Kagome Cavities
Source: Adv Mater. 2025 Dec 9;38(9):e14310. doi: 10.1002/adma.202514310 (PMC12902589; doi:10.1002/adma.202514310)
Supplement: Supplementary file 1 — Supporting Information [file ADMA-38-e14310-s001.pdf]

ADVANCED MATERIALS

Supporting Information

for *Adv. Mater.*, DOI 10.1002/adma.202514310

# Circularly Polarized Polariton Lasing from Spin-Momentum Locking in Deformed Plasmonic Kagome Cavities

*Zhaoyun Zheng, Chuchuan Hong, Siamak Khorasani, Shreya K. Patel, Marc R. Bourgeois, David J. Masiello* and Teri W. Odom**

**Supporting Information**

# Circularly Polarized Polariton Lasing from Spin-momentum Locking in Plasmonic Kagome Cavities

Zhaoyun Zheng,[a,¶] Chuchuan Hong,[a,¶] Siamak Khorasani,[c] Shreya K. Patel,[a]
Marc R. Bourgeois,[d] David J. Masiello,[c,d,*] and Teri W. Odom [a,b,*]

a. Department of Chemistry, Northwestern University, Evanston, Illinois 60208, United States.
b. Department of Materials Science and Engineering, Northwestern University, Evanston, Illinois 60208, United States.
c. Department of Materials Science and Engineering, University of Washington, Seattle, Washington 98195, United States
d. Department of Chemistry, University of Washington, Seattle, Washington 98195, United States

¶ Authors contributed equally

* Corresponding authors: masiello@uw.edu; todom@northwestern.edu

## Table of Contents

**1. Modified Photonic Band Structure through $K_R$-corrected Empty lattice Approximation ........ S3**

**2. FDTD Simulations of Mode Splitting at K and T points ........ S6**

**3. SEM Images of Deformed Kagome Lattice with Reduced Periodicity ........ S8**

**4. Circular Dichroism Simulation near K points in Deformed Kagome Lattice ........ S9**

**5. Group Theory and Mode Analysis at T point in Deformed Kagome Lattice ........ S10**

**6. Mode Analysis and Dipole Distribution at T points in Undeformed Kagome Lattices ........ S13**

**7. Optical Characterization of 4-monolayer CdSe nanoplatelets ........ S15**

**8. Coupled Oscillator Model and FDTD Dispersion Simulation ........ S16**

**9. FDTD Simulation of Waveguiding Effects ........ S19**

**10. Fourier space DCP map at T-points ........ S20**

**11. Fluence-dependent angle-resolved polariton emissions measurements ........ S21**

**12. Light Cone Analysis ........................................................................................ S22**

**13. Polariton Lasing from K points ........................................................................ S25**

**14. $S_3$ Parameters of T-point Polariton Lasing in Undeformed Kagome Lattices............. S27**

**15. SLR Eigenmodes – Coupled Dipole Approach ................................................ S28**

**16. Supporting Information References.................................................................... S30**

## 1. Modified Photonic Band Structure through $K_R$-corrected Empty Lattice Approximation

How unit-cell geometry design in real space influences reciprocal space can be generalized using a $K_R$-corrected empty lattice approximation (ELA), where an additional momentum vector $\pm \alpha_R K_R$ is added to the reciprocal lattice vectors $G$:

$$|k_{//} + G_{//} \pm \alpha_R K_R| = |k_{inc}| \qquad \text{Eq. S1}$$

where $k_{//}$ is the in-plane wavevector of incident light, and $G_{//}$ the reciprocal vector defined by the lattice. $k_{inc} = nE / \hbar c$ is the wavenumber of incident light, and $E$ is the energy of the photon, $n$ the refractive index of the medium, $\hbar$ the reduced Planck's number, and $c$ the speed of light in free space. $K_R$ is associated with the geometric phase, which describes the interplay between phase shifts and polarization changes as light passes through the optical structures. The magnitude of $K_R$ is defined by $\alpha_R$, and the direction is determined by the lattice design. The $\pm$ sign in front of $\alpha_R$ is determined by the spin angular momentum of photons, i.e., the handedness of circular polarization defined by the Optical Rashba Dresselhaus (ORD) effect.[1, 2]

For Kagome lattices, we use the notation from published work and set $K_R$ perpendicular to $G$.[3, 4] The values of $\alpha_R$ in deformed Kagome lattices depend on shrinking ($a_0$ – s, yellow triangles) and expanding ($a_0$ +s, green triangle) the trimer unit cell while maintaining the lattice periodicity $A_0$ (**Figure S1a**). We treated $\alpha_R$ as a fitting parameter to adjust the dispersion to match both the experimental transmission and numerically simulated band structure.

Due to the addition of $\pm \alpha_R K_R$, the original diffraction orders (black dots) rotate clockwise or counterclockwise (**Figure S1b**). The blue circles represent modified diffraction orders by $+ \alpha_R K_R$, while the red circles correspond to those shifted by $- \alpha_R K_R$; photons with opposite circular polarization are deflected to distinct locations in Fourier space. The calculated modes using $K_R$

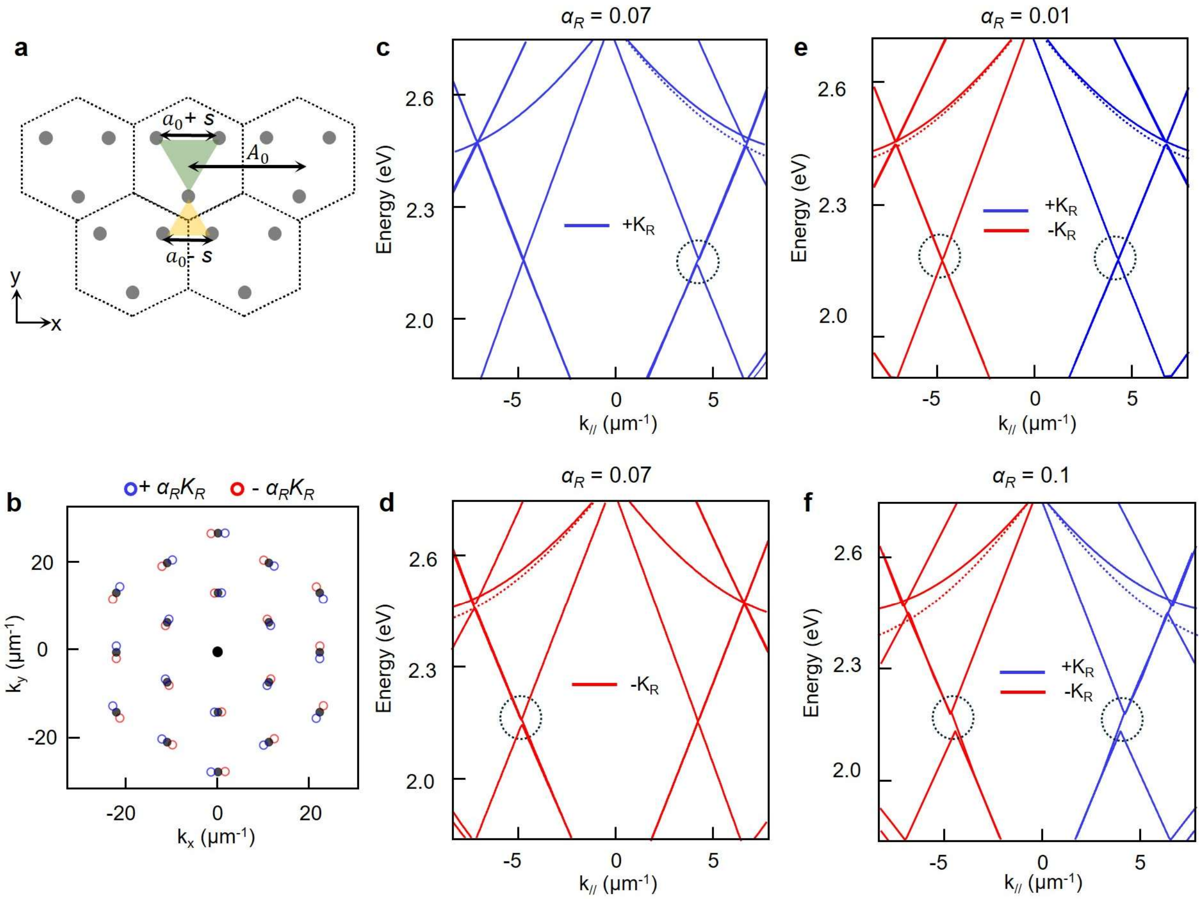


**Figure S1: $K_R$ -corrected empty lattice approximation. (a)** Scheme of deformed lattice. Yellow and green triangles indicate shrunken ($a_0$ - $s$) and expanded ($a_0$ + $s$) trimer unit cells, respectively, while the overall lattice constant $A_0$ = 580 nm remains the same as in the undeformed Kagome lattice. **(b)** Reciprocal space showing 1st and 2nd order diffraction modes (black dots) and distortions after $+K_R$ (blue circle) and $–K_R$ (red circle) corrections. G and G′ are the original and corrected reciprocal lattice vectors. **(c)** To account for the photonic band structure changes in deformed Kagome lattices, ELA with $+K_R$ modified the dispersion along positive wavevectors and **(d)** ELA with $–K_R$ modified the dispersion along negative wavevectors. **(e)** The ELA with a small $\alpha_R$ value (~ 0.01) results in only minor modifications to the dispersion, with the emergence of additional parabolic bands (dotted lines) and the band gap opening (dotted circles). **(f)** A larger $\alpha_R$ value (~0.1) induces more significant changes to the mode splitting.

with opposite signs correspond to the eigenstates with different circular polarizations. With $\alpha_R$ ~ 0.07, **Figure S1c** shows that $+K_R$ modifies bands at positive wavevectors, as highlighted by the additional parabolic mode (dotted lines) and band gap opening (dotted circle), while $-K_R$ affects those at negative wavevectors (**Figure S1d**). Moreover, the extent of band modification increases

with larger $\alpha_R$, as evident from the greater energy splitting between the parabolic bands and the larger band gap openings when comparing $\alpha_R \sim 0.1$ (**Figure S1e**) and $\alpha_R \sim 0.01$ (**Figure S1f**). Since ELA does not consider interactions among nanoparticles, the degree of circular polarization and magnitude of mode splitting are best represented by numerical simulations such as FDTD and the coupled dipole approximation.

## 2. FDTD Simulations of Mode Splitting at K and T points

To complement the $K_R$-corrected ELA, we conducted finite-difference time-domain (FDTD) simulations (Ansys-Lumerical) to examine how modifications to the trimer nanoparticle spacing (*s*) influenced transmission at the T and K point of Al Kagome lattices. The transmission spectra were calculated using a TE-polarized plane wave source with incident angle $\theta \approx 18°$ for the T point in air and $\theta \approx 23°$ for the K point. For simulated Kagome lattices, we used a lattice periodicity of $A_0$ = 580 nm, Al nanoparticles with diameter $d$ = 80 nm and height $h$ = 55 nm and varied the unit cell deformation *s* from 0 to 90 nm. In **Figure S2a**, the undeformed lattice (*s* = 0 nm, blue line) exhibits a single resonance mode at E ≈ 2.18 eV for the T point. As *s* increased, the single SLR mode split into two distinct modes, with the energy separation between them also increasing. The transmission intensity between modes is not equivalent: the lower-energy mode exhibited higher intensity than the higher-energy one. A similar trend was observed at the K point, where increasing *s* caused the original single resonance at 2.45 eV to divide into two modes. With increasing *s*, the

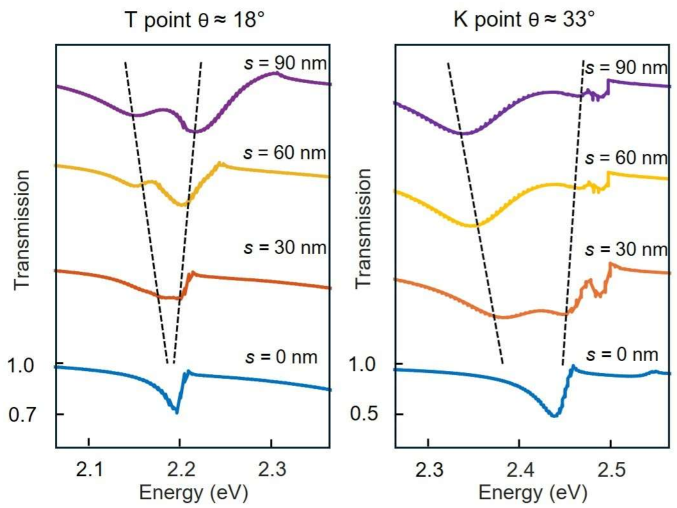


**Figure S2: Mode splitting at T and K points. (a)** FDTD simulated transmission spectra of Al Kagome lattices illuminated with a TE-polarized plane wave source at the T point ($\theta \approx 18°$) with increasing change in trimer unit cells from 0 nm to 90 nm. **(b)** Simulated transmission spectra at the K point ($\theta \approx 23°$) with the same excitation source and lattice parameters. The changes of mode energies with different *s* values are tracked by the dashed lines.

higher-energy mode gradually shifts toward and approaches the Rayleigh anomaly at 2.49 eV (**Figure S2b**). The simulated transmission spectra show larger mode splitting at both T and K points with larger $s$, which is consistent with the trend in the $K_R$-corrected ELA for increasing $\alpha_R$, which confirms a positive correlation between the real-space deformation and the magnitude of $K_R$ in reciprocal space.

## 3. SEM Image of Deformed Kagome Lattice with Reduced Periodicity

**Figure S3** shows a fabricated deformed Kagome lattice with periodicity ($A_0$ = 500 nm, $a_0$ - $s$ = 175 nm, $a_0$ + $s$ = 325 nm, $d$ = 70 nm and $h$ = 55 nm) designed to couple the T-points and the heavy-hole excitons of 4-ML CdSe nanoplatelets. Nanoparticle dimensions were chosen to ensure sufficient scattering from Al nanoparticles while preserving high quality factors (Q) of the cavity modes for lasing.

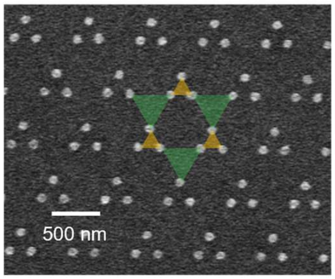


**Figure S3: SEM image of a fabricated lattice with Al NPs.** Yellow and green triangles refer to the trimer unit cells of the deformed Kagome lattice.

## 4. Circular Dichroism Simulation near K points in Deformed Kagome Lattices

**Figure S4** shows the FDTD-calculated CD transmission band structure of a deformed Kagome lattice based on lattice parameters $A_0$ = 580 nm, $a_0 - s$ = 203 nm, $a_0 + s$ = 377 nm, $d$ = 80 nm and $h$ = 55 nm. For this configuration, the K point resonance ($k_{//}$ = ± 7.0 $\mu m^{-1}$ and $E$ = 2.45 eV) is close to the heavy-hole exciton of 4-ML CdSe nanoplatelets. Here, the relative magnitude of CD (± 0.35) is lower than that of T-points at similar energies (± 0.59) in Figure 3a.

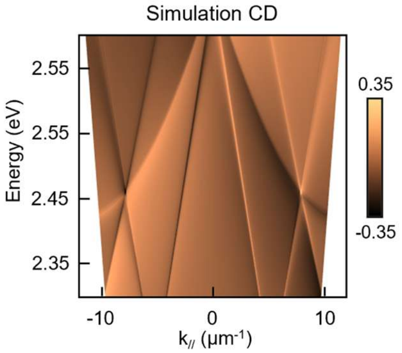


**Figure S4: Simulated circular dichroism (CD) in deformed Kagome lattices.** The calculated CD at the K points has values of ± 0.35.

**5. Group Theory and Mode Analysis at T point in Deformed Kagome Lattice**

The transition from linearly polarized to elliptically polarized fields upon breaking inversion symmetry of the Kagome lattice can be intuitively understood from group theory. The unbroken Kagome structure is characterized by $6/mmm$ ($D_{6h}$) crystallographic symmetry. At any six T points in reciprocal space, $G_{\boldsymbol{K}}/T = C_{2v}$. The irreducible representations (irreps) of the six in-plane polarized SLRs are $3A_1 + 3B_2$, which can be transformed as Cartesian unit vectors oriented parallel ($\hat{x}$) and perpendicular ($\hat{y}$) to the reciprocal vector, respectively. The separation of in-plane polarized SLRs into two orthogonally polarized classes underlies the linear polarization of the far-field light radiated by the undeformed Kagome lattice SLRs. The deformed Kagome structures are characterized by $\overline{6}m2$ ($D_{3h}$) crystallographic symmetry due to the absence of inversion symmetry. At any of the T points, $G_{\boldsymbol{K}}/T = C_s$, all six in-plane polarized SLRs are associated with the $A'$ irrep. Hence, (i) there are no degeneracies of the modes, and (ii) SLRs are generally elliptically polarized.

Predictions derived from the symmetry analysis of Kagome lattices can be further corroborated by eigenenergy calculations using the coupled-dipole method, which provides a direct comparison of the dispersion and near-field dipole polarization changes under lattice deformation. **Figure S5a** shows eigenmodes at and near the T points for a deformed Kagome lattice ($A_0$ = 500 nm, $\Delta s$ = 75 nm) and nanoparticles with $d$ = 70 nm and $h$ = 55 nm. Circles denote the calculated eigenmodes, with circles of larger size and lighter color representing the SLR modes of higher quality factor. The black solid lines are drawn as a guide to represent the SLR dispersion that crosses at the T point around $k_{//}$ = -5.6 μm$^{-1}$, with the energy separation between the upper and lower SLR branches ca. 40 meV. At $k_{//}$ = -5.6 μm$^{-1}$, we identify three SLR modes in total: one at 2.53 eV around the minimum of the upper branch, one at 2.52 eV along the linear dispersion, and one at 2.49 eV within

the maximum of the lower SLR branch. Quasinormal-mode analysis allows us to assign the irreducible representations to these three modes based on their mode symmetry in real space; all three belong to the same representation ($A'$) (**Figure S5b**). **Figure S5c** shows the time-averaged field map for the trimer unit cell at 2.53 eV with the highest Q factor. The electric field representing dipolar localized plasmons is tightly confined close to the nanoparticle surface. Notably, the dipoles are oriented along different directions, which represents the phase delay of electric fields among nanoparticles that support spin polarization at the T points.

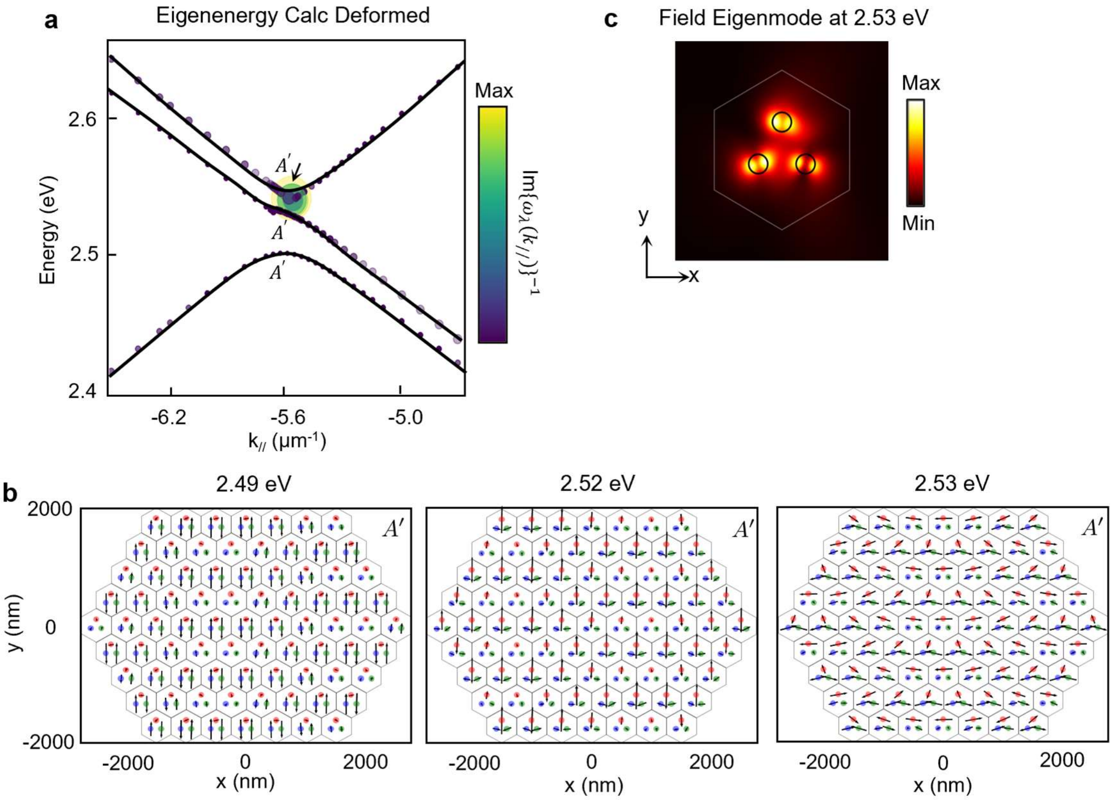


**Figure S5: Mode analysis at the T point for deformed Kagome lattices. (a)** Coupled dipole eigenmode dispersion near the T point for a deformed Kagome lattice with marker colors representing Q factors. The highest-quality mode at the T point ($k_{//}$ = - 5.6 μm$^{-1}$ and 2.53 eV) is pointed by the arrow. The black lines are visual guides to the eigenmode dispersion. **(b)** T point SLR mode symmetries ($G_K/T = C_s$) associated with a deformed Kagome lattice. 10 × 10 unit cells are shown here to display the spatial extent of the mode near T points at each eigenenergy. **(c)** Eigenmode field map at 2.53 eV shows electric fields that are tightly confined around the nanoparticles and oriented in different directions.

## 6. Mode Analysis and Dipole Distribution at T points in Undeformed Kagome Lattices

**Figure S6a** shows the same eigenmode calculations at and near the T points for an undeformed Kagome lattice ($A_0$ = 500 nm, $a_0$ = 250 nm) and nanoparticles with $d$ = 70 nm and $h$ = 55 nm. The hybridization of diffractive modes (grey dotted lines) with the localized surface plasmons (LSPs) of Al nanoparticles produced a linear band along the (0-10)/(00-1) direction. In addition, at $k_{//}$ = - 5.6 μm$^{-1}$, the upper and lower SLR branches now have an energy gap less than 20 meV, with the linear band nearly overlapping with the lower SLR branch. As shown in **Figure S6b**, the SLR

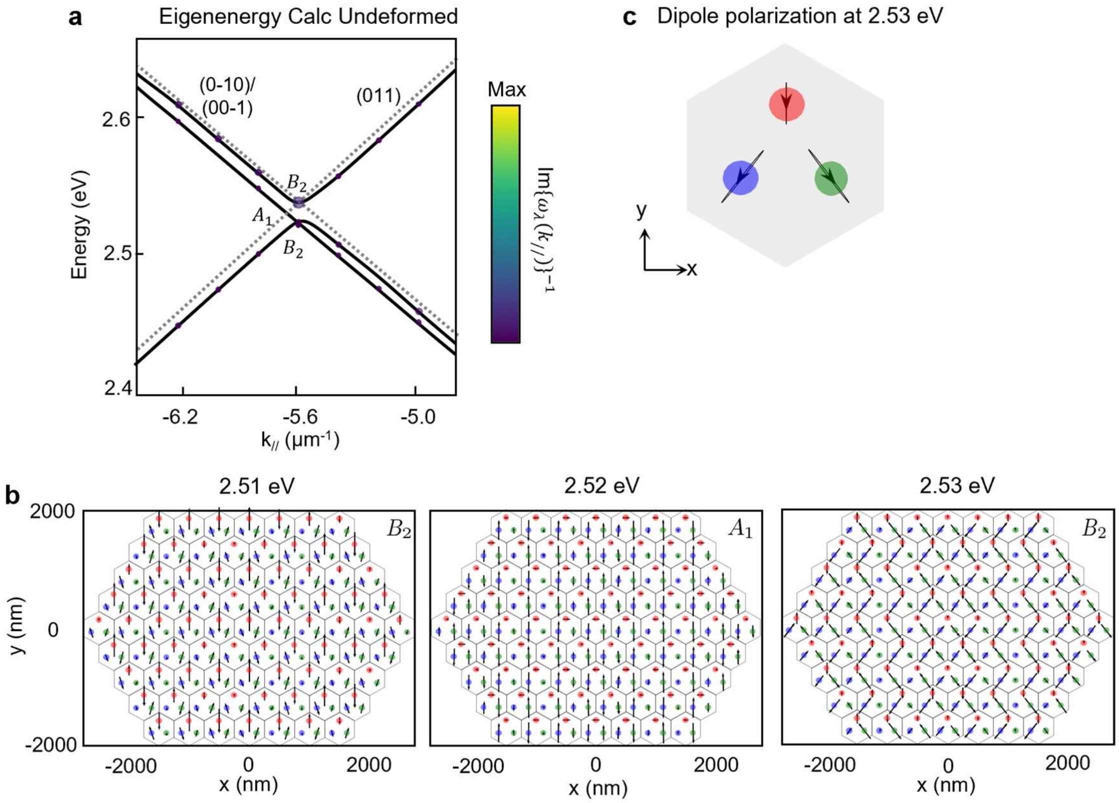


**Figure S6: Mode analysis at the T point for an undeformed Kagome lattice. (a)** Coupled dipole eigenmode dispersion near the T point for an undeformed Kagome lattice with marker colors representing Q factors derived from the imaginary part of the eigenfrequencies. The highest-quality mode at the T point ($k_{//}$ = - 5.6 μm$^{-1}$ and 2.53 eV) are highlighted by the arrow. The light grey dotted line shows the dispersion from the empty lattice approximation, while the black solid lines serve as visual guides to the eigenmode dispersion. The dipole polarizations within a unit cell of the undeformed lattice are linear. **(b)** T point SLR mode symmetries ($G_K/T = C_{3v}$) associated with an undeformed Kagome lattice. **(c)** The dipole moments in the deformed lattice at 2.53 eV exhibit linear polarization structures.

eigenmodes at 2.53 and 2.51 eV both belong to the $B_2$ representation, whereas the diagonal mode in between at 2.52 eV corresponds to the $A_1$ representation. Unlike in the deformed Kagome lattice case where all modes had the same representation ($A'$), the two distinct irreps here are orthogonal, indicating that there is no interaction between these SLR modes. Also, in undeformed Kagome lattices, the unit-cell dipole distributions associated with the T-point band edge (2.53 eV) are predominantly linear (**Figure S6c**), which supports the lack of spin components in the cavity resonance.

## 7. Optical Characterization of Four-monolayer CdSe Nanoplatelets

**Figure S7** illustrates the measured absorption and photoluminescence. The heavy-hole (HH) exciton absorption and emission spectrum both have linewidths ~50 meV. Such narrow linewidths indicate reduced dephasing rate and high optical gain of the synthesized emitters.[S5-7]

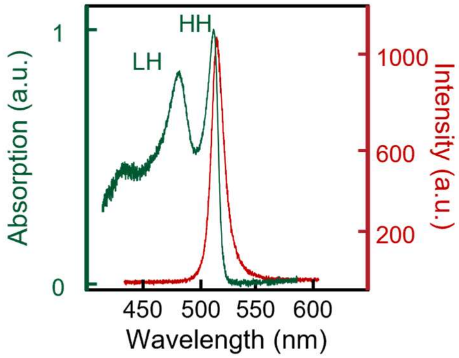


**Figure S7: Optical characterization of CdSe nanoplatelet films** (thickness = 120 nm).

## 8. Coupled Oscillator Model and FDTD Dispersion Simulation

To determine the Rabi splitting of our system, we used a coupled-oscillator model to fit the exciton-polariton dispersion.[8-10] The calculation involved (1) computing the uncoupled cavity dispersion, and (2) calculating the polariton branches. In the first step, the energy of the SLR modes was approximated using Rayleigh anomaly (RA) dispersions derived from the $K_R$-corrected empty lattice approximation with $A_0 = 500$ nm (**Figure S8a**).

A thin film of CdSe nanoplatelets on glass substrates can generate waveguiding effects and modify the effective refractive index surrounding the nanoparticle lattice, with the waveguide mode dispersions of various polarizations described as:[S11, 12]

For TM-polarized waveguide mode: $\tan(\kappa t) = \frac{\kappa(\gamma+\delta)}{\kappa^2-\gamma\delta}$ Eq. S2

For TE-polarized waveguide mode: $\tan(\kappa t) = \frac{n_w^2\kappa(n_s^2\gamma+\delta)}{n_s^2\kappa^2-n_w^2\gamma\delta}$ Eq. S3

Effective index of a waveguide mode: $n_{eff} = \frac{\beta}{k_0}$ Eq. S4

where $\kappa = \sqrt{n_w^2 k_0^2 - \beta^2}$, $\gamma = \sqrt{\beta^2 - n_s^2 k_0^2}$, $\delta = \sqrt{\beta^2 - k_0^2}$.

$n_w$ and $n_s$ are the refractive indices of the NPL layer and the glass substrate, respectively. t = 120 nm is the thickness of the NPL film layer;[8] $k_0$ is the wavenumber in free space; $\beta$ is the propagation constant in the waveguide, which can be considered as the in-plane momentum added to the diffractive modes due to the waveguide modes. Their values can be numerically solved using Eqns. S2-S4. Although the high-index films can support multiple waveguide modes with different effective refractive indices, we used the average of the fundamental TE- and TM-mode with $n_{eff}$ = ~1.675 since the values are nearly constant across the wavelength range of interest (**Figure S8b**).

Using the calculated effective index, the T points at $k_{//} = \pm 5.6\ \mu m^{-1}$ and 2.33 eV (dotted circles)

were negatively detuned from the HH exciton band at 2.42 eV (green dotted line). Polaritonic dispersions were obtained by solving the eigenenergies of a coupling matrix describing energy exchange between heavy-hole (HH) excitons in the CdSe NPLs and the cavity modes:

$$\begin{pmatrix} E_{RA}(k_{//}) & g \\ g & E_{HH} \end{pmatrix} \quad \text{Eq. S5}$$

**Figure S8c** presents the calculated band structure with the Rabi splitting $g$ = 75 meV, determined at the crossing between the HH exciton at 2.42 eV (green dotted line) and the cavity modes (light gray lines). The polariton dispersion shows good agreement with the measured

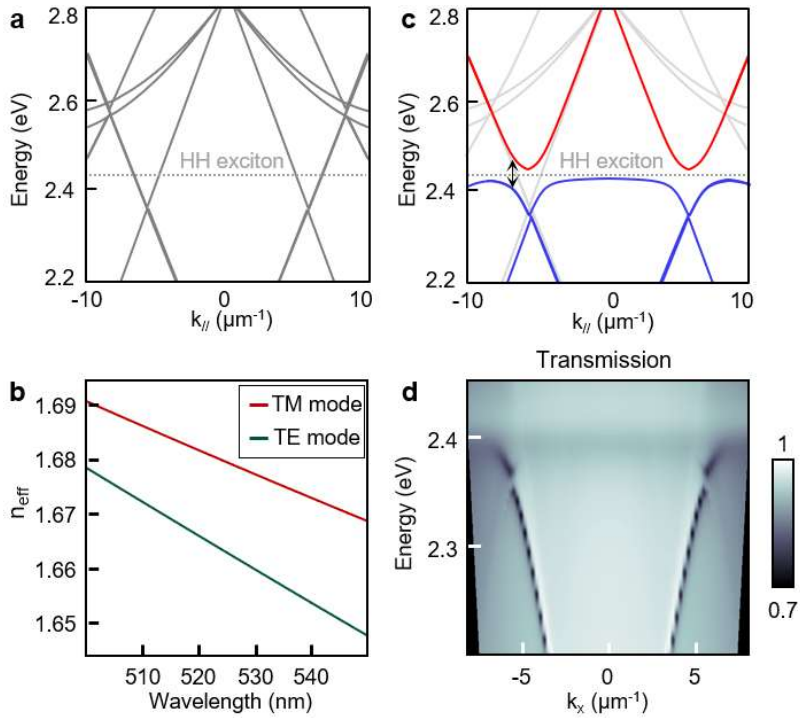


**Figure S8: Coupled-oscillator modeling confirming strong coupling. (a)** Calculated empty lattice dispersion of a Kagome lattice with periodicity of $A_0$ = 500 nm with $n_{eff}$ =1.675. The HH exciton at 2.42 eV is marked by the green dotted line, and the positions near T-point by dotted circles. **(b)** Effective refractive index of fundamental TE and TM waveguide modes near T-points. **(c)** Calculated polariton bands from strong coupling between HH excitons (green dotted line) and T-point SLRs. The uncoupled dispersions are highlighted with gray lines. The blue curves represent the lower polariton branch, while the red curves denote the middle polariton branch. The energy separation between these two polaritonic branches yields a Rabi splitting ($g$) of 75 meV at the intersection point between the cavity mode and the HH exciton (indicated by double arrows). **(d)** Numerically simulated band structures show the band splitting after integrating lattices with CdSe nanoplatelet films.

dispersion in Figure 4a, which validates our estimate of $g$. Although the light-hole (LH) exciton of CdSe nanoplatelets (at 2.58 eV), can also couple to the cavity to form middle and upper polariton branches, we only focused here on strong coupling of the HH exciton because the exciton energy is resonant with the T-point SLR mode. We compared the Rabi splitting with the average losses of the cavity and emitters to determine whether the system was in the strong-coupling regime. The calculated Rabi splitting $g = 75$ meV exceeds $(\gamma_{\mathrm{SLR}} + \gamma_{\mathrm{ex}})/2$, where $\gamma_{\mathrm{ex}} = 50$meV is obtained from the heavy-hole exciton linewidth of the CdSe nanoplatelet film and $\gamma_{\mathrm{SLR}} = 18$ meV is extracted from the angle-resolved transmission.

To support our analytical calculation (*i.e.* coupled oscillator model), we also conducted FDTD simulations using the experimental parameters of the deformed Kagome lattices and the CdSe nanoplatelets film of 120 nm with the optical properties obtained from the ellipsometry.[13] **Figure S8d** shows the band splitting expected around T points as well as the anticrossing behavior close to the HH (2.42 eV).

## 9. FDTD Simulation of Waveguiding Effects

Contributions of waveguiding effects were examined using FDTD simulations of the near-field distribution for Al lattices combined with CdSe nanoplatelet films. The film was modeled as a non-absorbing dielectric slab with a uniform refractive index of $n = 1.83$ and a thickness of 120 nm based on prior studies using similar synthesis and spin-coating conditions.[14] A TE-polarized plane wave at an incident angle of 18° was used as the excitation source, and the field monitor was set to a wavelength of 525 nm. **Figure S9a** shows an asymmetric electric field distribution in the cross section because excitation is at an oblique angle. The fields are concentrated primarily around the nanoparticles but also extend into the surrounding film. **Figure S9b** depicts a phase reversal at the air-film interface (most prominent in the boxed region), indicating that light is trapped inside the high-index film due to waveguiding effects.[8, 11, 15, 16]

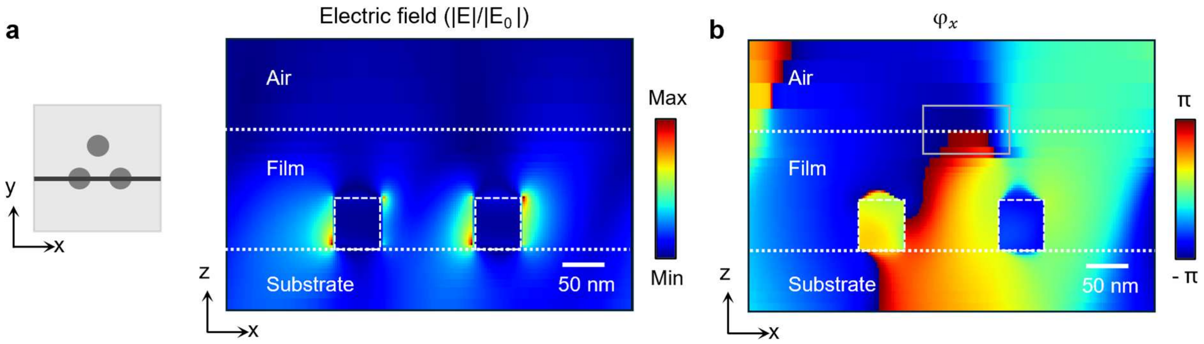


**Figure S9: FDTD simulations of waveguiding effects. (a)** (left) Scheme showing the position of the field monitor (black line) at the center between two nanoparticles within the trimer unit cell. This monitor is used in simulations to extract the field distribution along the xz-plane. (right) The field is mainly localized around the nanoparticles, with some extension into the surrounding film. **(b)** The phase flip observed at the air-film interface provides evidence for waveguiding effects.

## 10. Fourier space DCP map at T-points

**Figure S10** shows the measured DCP map in Fourier space around the energy level of T points (~2.32 eV). The DCP values of ~ ± 0.6 agree well with those from the angle-resolved measurement. Moreover, the alternating positive and negative regions every 60° are consistent with the group theory predictions for deformed Kagome lattices, where spin polarization is tied to the discrete rotational symmetry. We note that the pattern is slightly distorted and the edges of the hexagon appear broad and blurred due to the finite bandwidth of the band-pass filter (~ 10 nm).

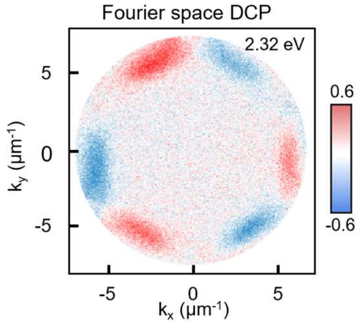


**Figure S10: DCP map in Fourier space**. The values match angle-resolved measurement, and the alternating negative/positive values follows the estimation from the mode analysis using dipole interactions.

## 11. Fluence-dependent angle-resolved polariton emissions

**Figure S11** shows the angle-resolved photoluminescence around T-point lower polariton under various pump fluence. Below lasing threshold, the emission linewidth is ~ 6 nm; at threshold (~8 μJ/cm$^2$), a bright emission spot emerged at T-point with narrow linewidth (~0.4 nm) due to the coherency of emission. As pump fluence keeps increasing, the emission shows a blue-shifting center wavelength due to the polariton-polariton and polariton-exciton repulsive interactions.

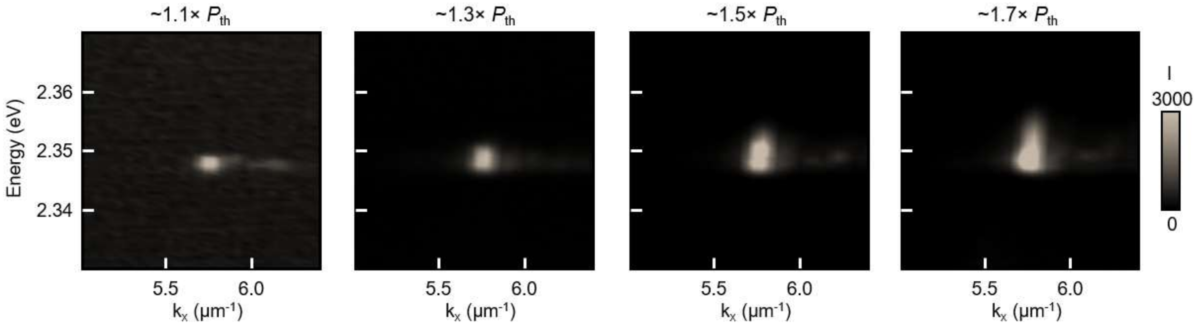


**Figure S11: Angle-resolved lasing beams at T-point under various pump fluence.** As lasing fluence increases from $P_{th}$ to 1.6× $P_{th}$, the center wavelength of the polariton lasing from T-point blue shifts due to polariton-polariton or polariton-exciton repulsive interactions.

## 12. Light Cone Analysis

**Figure S12a** shows the Fourier plane photoluminescence map above threshold (1.1× $P_{th}$) from a deformed Kagome lattice ($A_0$ = 500 nm, $a_0 + s$ = 325 nm, $a_0 - s$ = 175 nm, $d$ = 70 nm and $h$ = 55 nm) and 4-monolayer CdSe nanoplatelet film of ~110 nm. The beam positions in Fourier space can be defined by two angular parameters in polar coordinates: polar angle (θ) and azimuthal angle (φ). θ represents the emission angle of lasing beams away from normal direction of the lattice plane, and φ is the in-plane azimuthal angle, where φ = 0 is defined as parallel to the positive $k_x$ direction. Several distinct emission features are observed including: (1) six bright spots at θ ≈ 18° and φ = 30, 90, 150, 210, 270 and 330° from three T and three T′ points; (2) six crosses at θ ≈ 25° and φ = 0, 60, 120, 180, 240 and 300°; and (3) twelve bright spots at θ ≈ 33°, with φ starting from 15° and spaced evenly with an angular interval of Δφ ≈ 30°. These lasing emission patterns can be explained using a light-cone model,[16, 17] where photons are outcoupled from the lattice via diffraction orders. At a given energy, the dispersion relation of emitted photons can be calculated using the equation: $|k_{\mathrm{mode}}| = En_{eff}/c\hbar$. where $n_{eff}$ = 1.68 is the effective refractive index based on the weighted average of the refractive index of the substrate ($n$ = 1.52) and the nanoplatelet film ($n$ = 1.80). **Figure S12b** illustrates the calculated mode distributions in Fourier space at the lasing emission energy $E$ = 2.33 eV. The first- and second-order diffraction orders are represented by red and blue dots, respectively, and the circles are the 2D projections of light cones formed by the diffractive modes.

In-plane waves can interfere at the intersection of the diffractive modes to provide optical feedback. The positions of these intersection points in reciprocal space ($k_x$ , $k_y$ ) can be converted to the real-space polar coordinates θ and φ that defines the measured radiation angle of the lasing

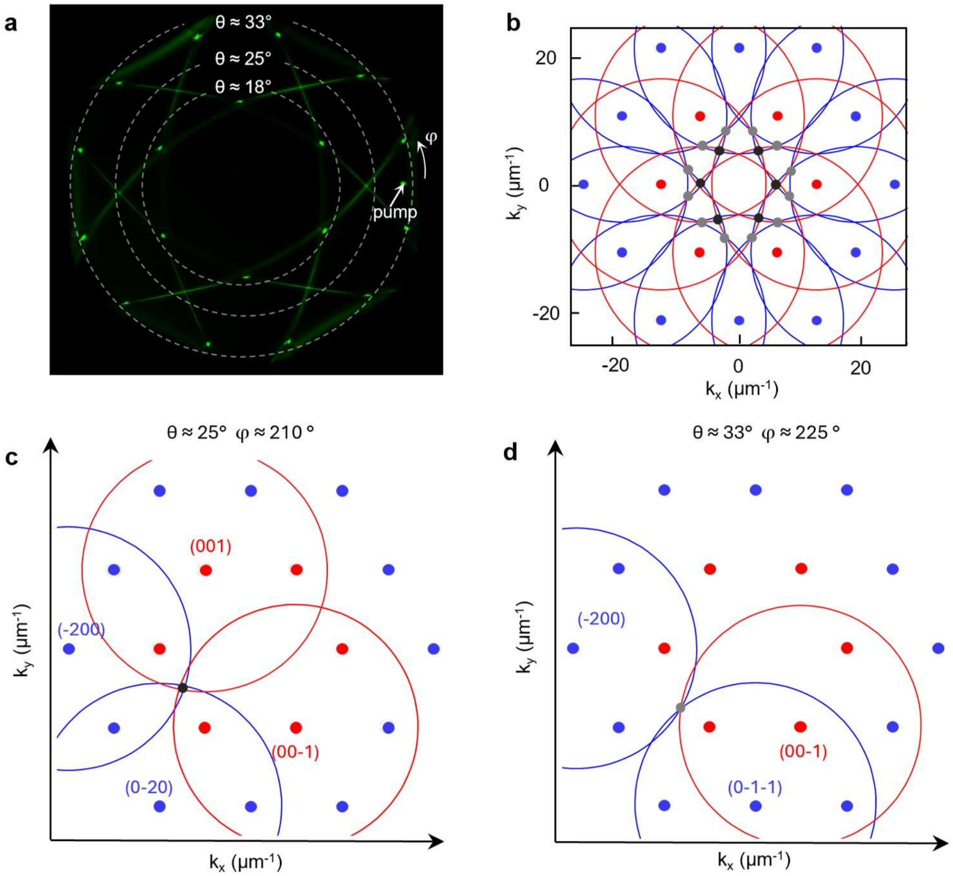


**Figure S12: Polariton lasing pattern in momentum space and light-cone analysis. (a)** Fourier plane image reveals six cross-shaped patterns at θ ≈ 25° and twelve bright spots at θ ≈ 33°. **(b)** Light-cone modeling at 2.33 eV with $n_{eff}$ = 1.68 shows first- and second-order diffraction orders as red and blue dots, with diffractive modes depicted as circles centered at these points. Grey and blue dots mark mode intersections where the photonic density of states was enhanced and where lasing were observed at these positions in experiment. **(c)** Diffractive modes contributing to the formation of a cross-shaped pattern at θ ≈ 25°. **(d)** Diffractive modes involved in forming one of the twelve bright spots at θ ≈ 33°.

emission. The cross shapes at θ ≈ 25° are formed by the intersection of two 1$^{st}$ and two 2$^{nd}$ order diffractive modes. For example, the 1$^{st}$ order modes (010) and (00-1) can intersect with 2$^{nd}$ order modes (-200) and (0-20) to form a cross-shaped pattern at φ = 210° (**Figure S12c**). The twelve bright spots observed at θ ≈ 33° are from intersections of one 1$^{st}$ (red) and two 2$^{nd}$ (blue) order diffraction orders; for example, a single bright spot can be formed from crossing of (00-1) and (0-

1-1) and (-200), respectively (**Figure S12d**). In both cases, these intersection points are determined by the six-fold rotational symmetry of the reciprocal lattice.

## 13. Polariton Lasing from K points

For K-point lasing, we used a deformed Kagome lattice with a larger periodicity ($A_0$ = 580 nm, $a_0 + s$ = 377 nm, $a_0 - s$ = 203 nm, $d$ = 80 nm and $h$ = 55 nm) to ensure that the K-point energy was isoenergetic with the heavy-hole exciton at 2.42 eV. **Figure S13a** presents the scattered photon distribution in Fourier space corresponding to the K-point diffraction orders at a lasing energy of 2.34 eV and $n_{eff}$ = 1.68. Three sets of energetically degenerate modes—two sets of second-order

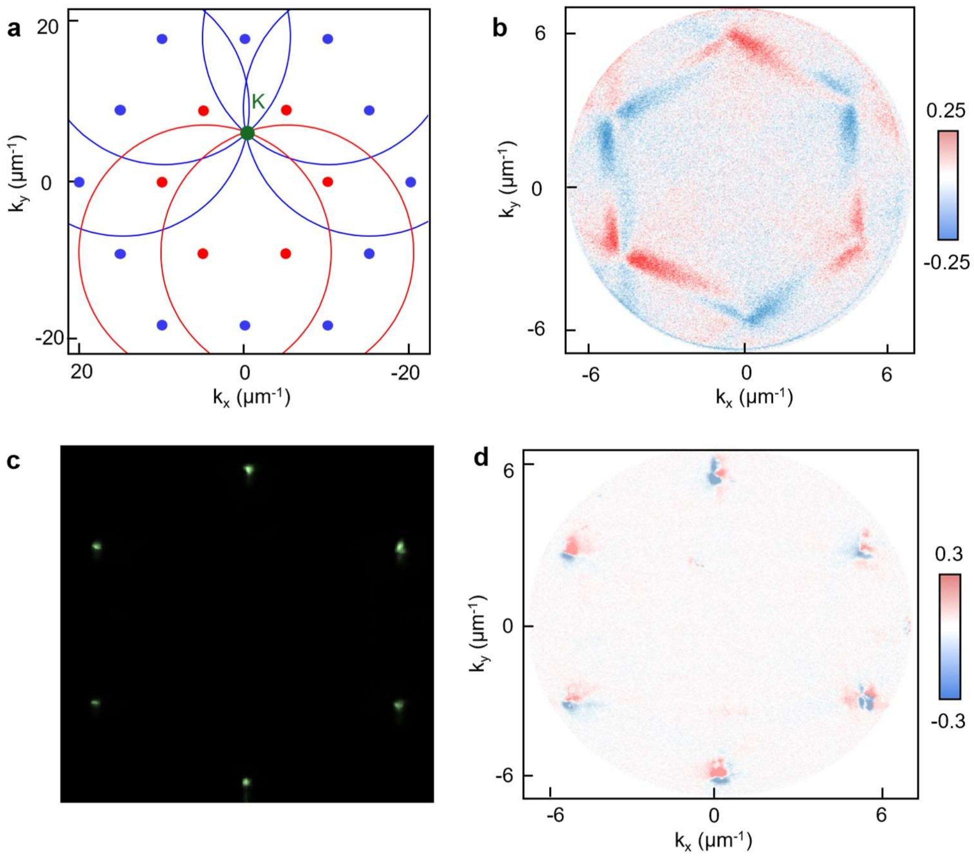


**Figure S13: Fourier plane images of polariton lasing from K points with deformed Kagome lattice. (a)** Light-cone analysis associated with the K point reveals the intersection of three sets of diffraction modes. **(b)** DCP map of K-point photoluminescence with alternating LCP and RCP emission at the vertices of the hexagon. **(c)** K-point lasing emission shows confined lasing beam profiles. **(d)** DCP map of K-point lasing shows overlapping LCP and RCP regions within a single beam spot, which is also reflected by the crossing of LCP and RCP modes observed in PL.

diffraction modes (e.g. (-101) / (110) and (020) / (002)) and one set of first-order diffraction modes (e.g. (00-1) / (0-10))—intersect at positions that define K points in momentum space.

**Figure S13b** displays the DCP of the photoluminescence associated with the K points in Fourier space. The DCP alternates between LCP (blue) and RCP (red) along the vertices of the hexagons, with a DCP value of ca. 0.35. Above the polariton lasing threshold, we observed confined bright spots at the K points (**Figure S13c**). Unlike beams from T-points with the elongated shapes, lasing emission from K/ K′ points is from high symmetry points in the Brillouin zone, and each of the lasing beams shows biaxially confined spots. **Figure S13d** shows that the K-point lasing beams have a DCP of ca. 0.4, which is similar to the DCP value observed in the photoluminescence and lower than that of T-point lasing. These differences can be attributed to interactions among nanoparticles within non-Bravais Kagome lattice unit cells; although K-points in honeycomb lattices can support circularly polarized eigenmodes,[18] the dipole strengths and orientations of the trimer unit cells in Kagome lattices can have a significant effect on the final polarization states in Fourier space.

## 14. $S_3$ Parameter of T-point Polariton Lasing in Undeformed Kagome Lattice

**Figure S14a** shows angle-resolved lasing emission at 1.5× $P_{th}$ from T points in an undeformed Kagome lattice ($A_0$ = 500 nm, $a_0$ = 250 nm, $d$ = 70 nm and $h$ = 55 nm) integrated with a ~110 nm CdSe NPL film. **Figure S14b** depicts that the DCP of the lasing beam in Fourier space had only minimal contrast at the T points (regions highlighted by dotted circles) compared to background. The low DCP value (± 0.1) likely emerges from extrinsic chirality when the lattice is excited at an oblique angle. [19-21] We note that lasing from T-points is not from high-symmetry band edges of Kagome lattices but from the intersections of diffraction orders.[15-17] Hence, we experimentally confirmed that circular polarization from T-points is very low when inversion symmetry is preserved.

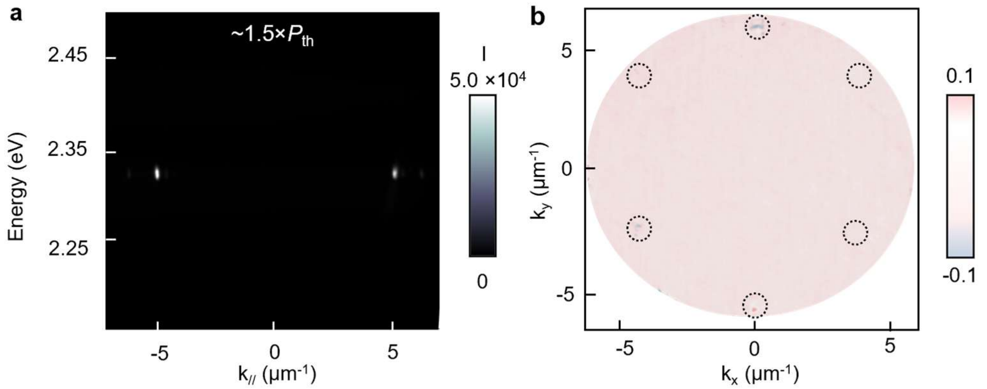


**Figure S14: T-point polariton lasing from undeformed Kagome lattice. (a)** Angle-resolved lasing emissions shows two distinct bright spots from T-point band edge from undeformed Kagome lattices. **(b)** DCP map of the T-point lasing shows minimal circular polarization.

## 15. SLR Eigenmodes – Coupled Dipole Approach

We considered a 2D Bravais lattices with $N_\kappa$ sites per unit cell, where the vector $\mathbf{x_n}$ denotes the center of the real space unit cell indexed by $n_1$and $n_2$, and $\mathbf{r}_\kappa$ is the displacement of site $\kappa \in \{1, \dots, N_\kappa\}$ relative to the center of the unit cell such that $\mathbf{x}_{\mathbf{n}\kappa} = \mathbf{x_n} + \mathbf{r}_\kappa$. A nanoparticle supporting localized surface plasmon excitations is located at each $\mathbf{x}_{\mathbf{n}\kappa}$. In the coupled dipole approximation, the electromagnetic response of each particle is characterized by the induced electric dipole $\mathbf{p}_{\mathbf{n}\kappa}(\omega) = \overline{\boldsymbol{\alpha}}_\kappa \mathbf{E}_{\mathbf{n}\kappa}(\omega)$, where $\overline{\boldsymbol{\alpha}}_\kappa(\omega)$ is the dipole polarizability tensor for sublattice $\kappa$. The total electric field at $\mathbf{x}_{\mathbf{n}\kappa}$ is $\mathbf{E}_{\mathbf{n}\kappa}(\omega)$ and is the sum of the incident $\mathbf{E}^0_{\mathbf{n}\kappa}$ and re-scattered fields from the other dipoles in the system, i.e.,

$$\overline{\boldsymbol{\alpha}}_\kappa^{-1} \cdot \mathbf{p}_{\mathbf{n}\kappa} = \mathbf{E}^0_{\mathbf{n}\kappa} + (-4\pi\omega^2) \sum'_{\mathbf{n}'\kappa'} \overline{\mathbf{G}}_0(\mathrm{x}_{\mathbf{n}\kappa}, \mathrm{x}_{\mathbf{n}'\kappa'}; \omega)\, \mathbf{p}_{\mathbf{n}'\kappa'}, \qquad \text{Eq. S6}$$

where the prime on the sum indicates the exclusion of the $\mathbf{n}' = \mathbf{n}$term when $\kappa' = \kappa$. Considering Bloch wave excitations of the form $\boldsymbol{\Theta}_{\mathbf{n}\kappa} = \boldsymbol{\Theta}_{\mathbf{K}\kappa} e^{i\mathbf{K}\cdot x_{\mathbf{n}}} e^{-i\omega t}$ with $\boldsymbol{\Theta} \in \{\mathbf{p}, \mathbf{E}_0\}$, the coupled dipole equation of motion in Eq. 6 can be expressed in terms of the Bloch wave coefficients

$$\begin{aligned}\overline{\boldsymbol{\alpha}}_\kappa^{-1} \cdot \mathbf{p}_{\mathbf{n}\kappa} &= \mathbf{E}^0_{\mathbf{n}\kappa} + \sum'_{\mathbf{n}'\kappa'} \overline{\mathbf{G}}_0(\mathrm{x}_{\mathbf{n}\kappa}, \mathrm{x}_{\mathbf{n}'\kappa'}; \omega) \cdot \mathbf{p}_{\mathbf{K}\kappa'} e^{i\mathbf{K}\cdot x_{\mathbf{n}'}} \\ &= \mathbf{E}^0_{\mathbf{n}\kappa} + \sum'_{\mathbf{n}'} \overline{\mathbf{G}}_0(\mathrm{x}_{\mathbf{n}\kappa}, \mathrm{x}_{\mathbf{n}'\kappa'}) \cdot \mathbf{p}_{\mathbf{K}\kappa'} e^{i\mathbf{K}\cdot x_{\mathbf{n}'}} + \sum_{\kappa'\neq\kappa} \sum_{\mathbf{n}'} \overline{\mathbf{G}}_0(\mathrm{x}_{\mathbf{n}\kappa}, \mathrm{x}_{\mathbf{n}'\kappa'}) \cdot \mathbf{p}_{\mathbf{K}\kappa'} e^{i\mathbf{K}\cdot x_{\mathbf{n}'}},\end{aligned} \qquad \text{Eq. S7}$$

which can be rewritten as

$$\left[\overline{\boldsymbol{\alpha}}_\kappa^{-1} - \sum'_{\mathbf{n}'} \overline{\mathbf{G}}_0(\mathrm{x}_{\mathbf{n}\kappa}, \mathrm{x}_{\mathbf{n}'\kappa'})\, e^{i\mathbf{K}\cdot(x_{\mathbf{n}'} - x_{\mathbf{n}})}\right] \cdot \mathbf{p}_{\mathbf{K}\kappa} - \sum_{\kappa'\neq\kappa} \sum_{\mathbf{n}'} \overline{\mathbf{G}}_0(\mathrm{x}_{\mathbf{n}\kappa}, \mathrm{x}_{\mathbf{n}'\kappa'})\, e^{i\mathbf{K}\cdot(x_{\mathbf{n}'} - x_{\mathbf{n}})} \cdot \mathbf{p}_{\mathbf{K}\kappa} = \mathbf{E}^0_{\mathbf{K}\kappa}. \qquad \text{Eq. S8}$$

Defining the $(3N_\kappa \times 1)$ vectors $\mathbf{p_K} = \sum_\kappa \overline{\overline{\mathbf{I}}}_{\kappa\kappa} \mathbf{p}_{\mathbf{K}\kappa}$ and $\mathbf{E}^0_{\mathbf{K}} = \sum_\kappa \overline{\overline{\mathbf{I}}}_{\kappa\kappa} \mathbf{E}^0_{\mathbf{K}\kappa}$, where $\overline{\overline{\boldsymbol{I}}}_{\kappa\kappa}$ is the $(3N_\kappa \times 3N_\kappa)$ matrix of zeros except for the $(3 \times 3)$ identity matrix in the $\kappa$th diagonal block, the previous set of $N_\kappa$ coupled equations can be expressed as the single matrix equation

$$\overline{\overline{\Pi}}_{\mathbf{K}}(\omega) \mathbf{p_K} = \mathbf{E}^0_{\mathbf{K}}\,, \qquad \text{Eq. S9}$$

and the inverse of the $(3N_\kappa \times 3N_\kappa)$ SLR response matrix is

$$\bar{\bar{\Pi}}_{\mathbf{K}}(\omega) = \begin{pmatrix} \bar{\boldsymbol{\alpha}}_1^{-1} - \bar{\mathbf{S}}_{11}(\mathbf{K},\omega) & -\bar{\mathbf{S}}_{12}(\mathbf{K},\omega) & \cdots & -\bar{\mathbf{S}}_{1s}(\mathbf{K},\omega) \\ -\bar{\mathbf{S}}_{21}(\mathbf{K},\omega) & \bar{\boldsymbol{\alpha}}_2^{-1} - \bar{\mathbf{S}}_{22}(\mathbf{K},\omega) & & \vdots \\ \vdots & & \ddots & \\ -\bar{\mathbf{S}}_{s1}(\mathbf{K},\omega) & \ldots & & \bar{\boldsymbol{\alpha}}_s^{-1} - \bar{\mathbf{S}}_{N_\kappa N_\kappa}(\mathbf{K},\omega) \end{pmatrix}. \quad \text{Eq. S10}$$

The lattice $\bar{\mathbf{S}}_{\kappa\kappa}$ and interaction $\bar{\mathbf{S}}_{\kappa\kappa'}$ sum tensors, defined by

$$\bar{\mathbf{S}}_{\kappa\kappa}(\mathbf{K},\omega) = \textstyle\sum_{\mathbf{n}'}' \bar{\mathbf{G}}_0(\mathrm{X}_{\mathbf{n}\kappa}, \mathrm{X}_{\mathbf{n}'\kappa}, \omega)\, e^{i\mathbf{K}\cdot(x_{\mathbf{n}'} - x_{\mathbf{n}})} \quad \text{Eq. S11}$$

$$\bar{\mathbf{S}}_{\kappa\kappa'}(\mathbf{K},\omega) = \textstyle\sum_{\mathbf{n}'} \bar{\mathbf{G}}_0(\mathrm{X}_{\mathbf{n}\kappa}, \mathrm{X}_{\mathbf{n}'\kappa'}, \omega)\, e^{i\mathbf{K}\cdot(x_{\mathbf{n}'} - x_{\mathbf{n}})} \quad \text{Eq. S12}$$

account for intra- and inter-sublattice electromagnetic coupling, respectively.

Dispersive SLR (quasinormal) Bloch modes arise at $[\mathbf{K}, \omega_\lambda(\mathbf{K})]$ points where the matrix $\bar{\bar{\boldsymbol{\Pi}}}_{\mathbf{K}}^{-1}[\omega_\lambda(\mathbf{K})]$ becomes non-invertible, i.e., where $det(\bar{\bar{\boldsymbol{\Pi}}}_{\mathbf{K}}^{-1}[\omega_\lambda(\mathbf{K})]) = 0$. In the presence of material and radiative losses, $\bar{\bar{\boldsymbol{\Pi}}}_{\mathbf{K}}^{-1}[\omega_\lambda(\mathbf{K})]$ becomes non-Hermitian, and the mode energies become complex-valued with $Re\{E_\lambda(\boldsymbol{K})\} > 0$ and $Im\{E_\lambda(\boldsymbol{K})\} < 0$, where $E_\lambda(\boldsymbol{K}) = \hbar\omega_\lambda(\boldsymbol{K})$. A steepest descent method was employed to locate the relevant complex root regions by searching for zeros of the strictly non-negative function $f[E(\boldsymbol{K})] = |det\,(\bar{\bar{\boldsymbol{\Pi}}}_{\mathbf{K}}^{-1}[\omega_\lambda(\mathbf{K})])|$. The corresponding SLR Bloch mode polarization vectors $\boldsymbol{\epsilon}^\lambda[\mathbf{K}, \omega_\lambda(\mathbf{K})] = \sum_\kappa \bar{\bar{\mathbf{I}}}_{\kappa\kappa}\, \boldsymbol{\epsilon}_\kappa^\lambda\, [\mathbf{K}, \omega_\lambda(\mathbf{K})]$ spanning the null space of $\bar{\bar{\boldsymbol{\Pi}}}_{\mathbf{K}}^{-1}[\omega_\lambda(\mathbf{K})]$ were extracted from the singular value decomposition of $\bar{\bar{\boldsymbol{\Pi}}}_{\mathbf{K}}^{-1}[\omega_\lambda(\mathbf{K})]$ .

## Supporting Information References


(1) Zhou, J.; Xia, M.; Chen, Y.; Zhang, X. Circular-Polarization-Dependent Beam Deflection via Brillouin Zone Folding in Resonant Phase Gradient Metasurfaces. *ACS Photonics* **2024**, *11* (7), 2707-2712. DOI: 10.1021/acsphotonics.4c00553.

(2) Chen, Y.; Feng, J.; Huang, Y.; Chen, W.; Su, R.; Ghosh, S.; Hou, Y.; Xiong, Q.; Qiu, C.-W. Compact spin-valley-locked perovskite emission. *Nature Materials* **2023**, *22* (9), 1065-1070. DOI: 10.1038/s41563-023-01531-2.

(3) Shitrit, N.; Yulevich, I.; Kleiner, V.; Hasman, E. Spin-controlled plasmonics via optical Rashba effect. *Applied Physics Letters* **2013**, *103* (21). DOI: 10.1063/1.4832636 (acccessed 6/24/2025).

(4) Duan, X.; Wang, B.; Rong, K.; Liu, C.-l.; Gorovoy, V.; Mukherjee, S.; Kleiner, V.; Koren, E.; Hasman, E. Valley-addressable monolayer lasing through spin-controlled Berry phase photonic cavities. *Science* **2023**, *381* (6665), 1429-1432. DOI: doi:10.1126/science.adi7196.

(5) Gao, Y.; Weidman, M. C.; Tisdale, W. A. CdSe Nanoplatelet Films with Controlled Orientation of their Transition Dipole Moment. *Nano Letters* **2017**, *17* (6), 3837-3843. DOI: 10.1021/acs.nanolett.7b01237.

(6) Mack, T. G.; Jethi, L.; Kambhampati, P. Temperature Dependence of Emission Line Widths from Semiconductor Nanocrystals Reveals Vibronic Contributions to Line Broadening Processes. *The Journal of Physical Chemistry C* **2017**, *121* (51), 28537-28545. DOI: 10.1021/acs.jpcc.7b09903.

(7) Mazzotti, S.; Mühlematter, M. U.; Galli, G.; Norris, D. J. Theory of excitons in colloidal semiconductor nanoplatelets. *Physical Review B* **2024**, *110* (19), 195433. DOI: 10.1103/PhysRevB.110.195433.

(8) Hong, C.; Zheng, Z.; Patel, S. K.; Odom, T. W. High-Chirality Polariton Lasing from Symmetry-Broken Plasmonic Lattices. *ACS Nano* **2025**, *19* (19), 18824-18832. DOI: 10.1021/acsnano.5c04290.

(9) Freire-Fernández, F.; Sinai, N. G.; Hui Tan, M. J.; Park, S.-M.; Koessler, E. R.; Krauss, T.; Huo, P.; Odom, T. W. Room-Temperature Polariton Lasing from CdSe Core-Only Nanoplatelets. *ACS Nano* **2024**, *18* (23), 15177-15184. DOI: 10.1021/acsnano.4c03164.

(10) Sample, A. D.; Guan, J.; Hu, J.; Reese, T.; Cherqui, C. R.; Park, J.-E.; Freire-Fernández, F.; Schaller, R. D.; Schatz, G. C.; Odom, T. W. Strong Coupling Between Plasmons and Molecular Excitons in Metal–Organic Frameworks. *Nano Lett.* **2021**, *21* (18), 7775-7780. DOI: 10.1021/acs.nanolett.1c02740.

(11) Guan, J.; Sagar, L. K.; Li, R.; Wang, D.; Bappi, G.; Wang, W.; Watkins, N.; Bourgeois, M. R.; Levina, L.; Fan, F.; et al. Quantum Dot-Plasmon Lasing with Controlled Polarization Patterns. *Acs Nano* **2020**, *14* (3), 3426-3433. DOI: 10.1021/acsnano.9b09466.

(12) Guan, J.; Sagar, L. K.; Li, R.; Wang, D.; Bappi, G.; Watkins, N. E.; Bourgeois, M. R.; Levina, L.; Fan, F.; Hoogland, S.; et al. Engineering Directionality in Quantum Dot Shell Lasing Using Plasmonic Lattices. *Nano Lett* **2020**, *20* (2), 1468-1474. DOI: 10.1021/acs.nanolett.9b05342.

(13) Freire-Fernandez, F.; Sinai, N. G.; Hui Tan, M. J.; Park, S. M.; Koessler, E. R.; Krauss, T.; Huo, P.; Odom, T. W. Room-Temperature Polariton Lasing from CdSe Core-Only Nanoplatelets. *Acs Nano* **2024**, *18* (23), 15177-15184. DOI: 10.1021/acsnano.4c03164.

(14) Freire-Fernández, F.; Sinai, N. G.; Hui Tan, M. J.; Park, S.-M.; Koessler, E. R.; Krauss, T.; Huo, P.; Odom, T. W. Room-Temperature Polariton Lasing from CdSe Core-Only Nanoplatelets. *ACS Nano* **2024**. DOI: 10.1021/acsnano.4c03164.
(15) Guan, J.; Sagar, L. K.; Li, R.; Wang, D.; Bappi, G.; Watkins, N. E.; Bourgeois, M. R.; Levina, L.; Fan, F.; Hoogland, S.; et al. Engineering Directionality in Quantum Dot Shell Lasing Using Plasmonic Lattices. *Nano Lett.* **2020**, *20* (2), 1468-1474. DOI: 10.1021/acs.nanolett.9b05342.
(16) Tan, M. J. H.; Park, J.-E.; Freire-Fernández, F.; Guan, J.; Juarez, X. G.; Odom, T. W. Lasing Action from Quasi-Propagating Modes. *Advanced Materials* **2022**, *34* (34), 2203999. DOI: https://doi.org/10.1002/adma.202203999.
(17) Fasanelli, F. M.; Freire-Fernández, F.; Odom, T. W. Symmetry-Determined Lasing from Incommensurate Moiré Nanoparticle Lattices. *Advanced Optical Materials* **2024**, *12* (24), 2400797. DOI: https://doi.org/10.1002/adom.202400797.
(18) Yang, Y.; Jiang, H.; Hang, Z. H. Topological Valley Transport in Two-dimensional Honeycomb Photonic Crystals. *Scientific Reports* **2018**, *8* (1), 1588. DOI: 10.1038/s41598-018-20001-3.
(19) Lu, X.; Wu, J.; Zhu, Q.; Zhao, J.; Wang, Q.; Zhan, L.; Ni, W. Circular dichroism from single plasmonic nanostructures with extrinsic chirality. *Nanoscale* **2014**, *6* (23), 14244-14253, 10.1039/C4NR04433A. DOI: 10.1039/C4NR04433A.
(20) Cotrufo, M.; Osorio, C. I.; Koenderink, A. F. Spin-Dependent Emission from Arrays of Planar Chiral Nanoantennas Due to Lattice and Localized Plasmon Resonances. *ACS Nano* **2016**, *10* (3), 3389-3397. DOI: 10.1021/acsnano.5b07231.
(21) Liang, M.; Andreani, L. C.; Berghuis, A. M.; Pura, J. L.; Murai, S.; Dong, H.; Sánchez-Gil, J. A.; Gómez Rivas, J. Tailoring directional chiral emission from molecules coupled to extrinsic chiral quasi-bound states in the continuum. *Photon. Res.* **2024**, *12* (11), 2462-2473. DOI: 10.1364/PRJ.528976.
